# Exploring the Use of Alternative Promoters for Enhanced Transgene and sgRNA Expression in Atlantic Salmon Cells

**DOI:** 10.1007/s10126-024-10362-4

**Published:** 2024-08-30

**Authors:** Mohammad Ali Noman Reza, Thomas Nelson Harvey, Axmee Regmi, Jacob Seilø Torgersen, Guro Katrine Sandvik

**Affiliations:** 1 Department of Animal and Aquacultural Sciences, Norwegian University of Life Sciences, NO-1432 Ås, Norway; 2 AquaGen, NO-1432 Ås, Norway

**Keywords:** Atlantic salmon, CRISPR-Cas9, SgRNA expression, Transgene expression, Promoter activity, Endogenous promoters

## Abstract

This study facilitates design of expression vectors and lentivirus tools for gene editing of Atlantic salmon. We have characterized widely used heterologous promoters and novel endogenous promoters in Atlantic salmon cells. We used qPCR to evaluate the activity of several U6 promoters for sgRNA expression, including human U6 (hU6), tilapia U6 (tU6), mouse U6 (mU6), zebrafish U6 (zU6), Atlantic salmon U6 (sU6), medaka U6 (medU6), and fugu U6 (fU6) promoters. We also evaluated several polymerase type II (pol II) promoters by luciferase assay. Our results showed that hU6 and tU6 promoters were the most active among all the tested U6 promoters, and heterologous promoters (CMV, hEF1α core) had higher activity compared to endogenous Atlantic salmon promoters sHSP8, sNUC3L, sEF1α. Among endogenous pol II promoters, sEF1α and sHSP8 displayed higher activity than sNUC3L, sHSP703, sHSP7C, sXRCC1L, and sETF. We observed that extending the promoter sequence to include the region up to the start codon (ATG) resulted in a significant increase in expression efficiency for sNUC3L and sEF1α. We also show that mutating the PRDM1 motif will significantly decrease the activity of the sEF1α promoter. The presence of the PRDM1 motif in sHSP8 promoter was also associated with relatively high expression compared to the promoters that naturally lacked this motif, such as sNUC3L. We speculate that this short sequence might be included in other promoters to further enhance the promoter activity, but further experiments are needed to confirm this. Our findings provide valuable insights into the activity of different promoters in Atlantic salmon cells and can be used to facilitate further transgenic studies and improve the efficiency of transgene expression in Atlantic salmon.

**Supplementary Information:**

The online version contains supplementary material available at 10.1007/s10126-024-10362-4.

## Introduction

Atlantic salmon (*Salmo salar*) is an important food source for humans, and salmon farming is a rapidly growing industry. However, the sustainability of salmon aquaculture is facing challenges, especially with poor animal welfare caused by infectious diseases and parasites (Boyd et al. 2020). The Norwegian Fish Health Report from 2023 showed that 16.7% of farmed salmon died during the sea phase of the production period, and of these 38% were caused by infectious diseases. Major problems reported were winter sores, cardiomyopathy syndrome (CMS), gill disease, and heart and skeletal muscle inflammation (HSMI) (Sommerset et al. 2024). Genome engineering has the potential to address some of these challenges, by developing disease- and lice-resistant salmon (Blix et al. 2021; Gervais et al. 2021; Harvey et al. 2016; Lhorente et al. 2014; van Rijn et al. 2021). To accomplish this without adding foreign DNA sequences, endogenous expression systems must be developed and validated. Additionally, in vitro genetic manipulation is a powerful tool to identify the genes underlying desired traits, but tools for transgene expression are often developed in well-studied, distantly related species like human (*Homo sapiens*), mouse (*Mus musculus*), and zebrafish (*Danio rerio*) (Gervais et al. 2021; Pavelin et al. 2021). Although gene editing with plasmids has been accomplished in primary liver cells and SHK-1 cell line of Atlantic salmon (Datsomor et al. 2023; Strømsnes et al. 2022), there is to our knowledge no systematic comparison of endogenous and exogenous promoters in Atlantic salmon. In this study, we aimed to evaluate the activity of Polymerase type III (pol III) promoters, which are commonly used for driving expression of short non-coding RNA, such as gRNAs, and polymerase type II (pol II) promoters, which are essential for expression of protein coding genes.

The clustered regularly interspaced short palindromic repeats (CRISPR)—CRISPR associated protein 9 (Cas9) system has become a widely used technique for genome editing in Atlantic salmon in recent years. Plasmid-based CRISPR systems rely on the co-expression of two components in the target cells: a guide RNA (gRNA) and Cas. The gRNA will bind to Cas which makes a double-stranded cut in the DNA at the specific location decided by the gRNA sequence (Jinek et al. 2012). The expression of the gRNA is carried out by RNA polymerase III (pol III) in the cell. This polymerase is directed to pol III promoters such as the commonly used U6 promoter. The original function of U6 promoters in the organism is to drive expression of small nuclear RNA (snRNA) involved in RNA splicing (Didychuk et al. 2018). In contrast, polymerase II (pol II) promoters recruit RNA polymerase II and drive the expression of protein coding genes such as Cas9. Several studies demonstrate the inefficiency of CRISPR KO in fish cell lines when using human-based vectors (Escobar-Aguirre et al. 2019; Gratacap et al. 2020). Identifying better promoters that will ensure sufficient gRNA and Cas mRNA expression could potentially fix this problem, as previously shown in tilapia (*Oreochromis niloticus*) cells (Hamar & Kültz 2021).

An especially powerful application of in vitro genome editing is genome-wide CRISPR knockout screening which can be used to discover new candidate genes for disease resistance and other traits of interest (Shalem et al. 2015; T. Wang et al. 2014; Zhu et al. 2016). A crucial factor for the success of CRISPR screens in Atlantic salmon cells is identifying suitable promoters for driving the expression of gRNA and Cas9. Furthermore, because of the lentiviral vectors often used in genome-wide CRISPR screens are relatively large, and transfection efficiency can be negatively affected by vector size, the identification of minimal promoters would be beneficial.

While some studies have investigated the activity of potential promoters (zebrafish U6, fugu (*Takifugu rubripes*) U6, tilapia U6) activity in cell lines from other fish species (Boonanuntanasarn et al. 2009; Escobar-Aguirre et al. 2019; Hamar & Kültz 2021; Zenke & Kim 2008), knowledge about the activity of different promoters in Atlantic salmon cells is lacking. Next-generation salmon research will require access to a range of promoters with varying activity. In this study, we contribute to filling this knowledge gap by evaluating the activity of U6 promoters from six different species for sgRNA expression, in addition to four commonly used heterologous pol II promoters, and three novel endogenous pol II promoters of varying length.

## Materials and Methods

### Cell Culture

SHK-1 cells were obtained from the European Collection of Authenticated Cell Cultures (ECACC), and cultured according to previously described methods, with some modifications (Dannevig et al. 1997). To propagate Salmon head kidney (SHK-1) cells, a monolayer culture was established in Leibovitz L-15 medium (Sigma-Aldrich) with GlutaMAX™ (Gibco), 10% fetal bovine serum (Sigma-Aldrich), and 1% penicillin–streptomycin (Gibco). The cells were maintained at a temperature of 20 °C under ambient atmosphere and subcultured with 0.05% trypsin/EDTA (Sigma-Aldrich). Subculturing was performed when the cells reached 80–90% confluency, and the cells were passaged at a 1:2 ratio. All experiments were done using cells with a passage number less than 100 to minimize the risk of genetic instability and phenotypic changes (Geraghty et al. 2014). Furthermore, high passage numbers have been shown to be associated with changes in the cell properties in SHK-1 cells (Fourrier et al. 2007).

### Polymerase Type III Promoters

#### Identification, Characterization, and Analysis of the U6 Promoter

U6 promoters from human (hU6), mouse (mU6), zebrafish (zU6), medaka (*Oryzias latipes*; medU6), fugu (fU6), and tilapia (tU6) were obtained from the literature (Duvoisin et al. 2012; Escobar-Aguirre et al. 2019; Hamar & Kültz 2021; Roelz et al. 2010; Zenke & Kim 2008; see Supplementary Table S1 for NCBI Gene ID accession numbers, and Supplementary file S1 for sequences). For all U6 promoters included in the study, we identified regulatory regions using FIMO (Find Individual Motif Occurrences) analysis (Grant et al. 2011). This tool maps sequences of common pol III promoter elements such as the TATA box, CCAATYA, SPH, Octamer (OCT), and a proximal sequence element (PSE) derived from previously published U6 papers (Dahlberg & Lund 1988; Gelinas et al. 1985; Lifton et al. 1978; Schaub et al. 1999; Sturm et al. 1988). The potential matches were filtered by setting the *p*-value to *p* < 0.001. We looked for potential U6 promoters in the Atlantic salmon genome by selecting 247 bp upstream of the transcription start site of spliceasomal RNAs. To narrow down the list of salmon U6 promoters and to compare them to known U6 sequences from other species, we performed a motif analysis to identify the previously mentioned key U6-specific motifs. The salmon U6 with most key U6 motifs was selected for experimental analysis of promoter activity and named sU6 (NCBI Gene ID 123740086). Finally, a phylogenetic analysis of the different U6 promoters was performed utilizing the “build” function of ETE3 version 3.1.2 (Huerta-Cepas et al. 2016), on the GenomeNet platform (https://www.genome.jp/tools/ete/) to gain insights into their evolutionary relationship. The total sequence used for experimental purposes of each U6 promoter was used as input in the Multiple Sequence Alignment by CLUSTALW. ML tree was constructed using (PhyML v20160115) and ran with the following parameters: –pinv e –alpha e –nclasses 4 -o tlr -f m –bootstrap 100.

#### Production of Vectors with Different U6 Promoters for Evaluating gRNA Expression Efficiency

**Fig. 1:**
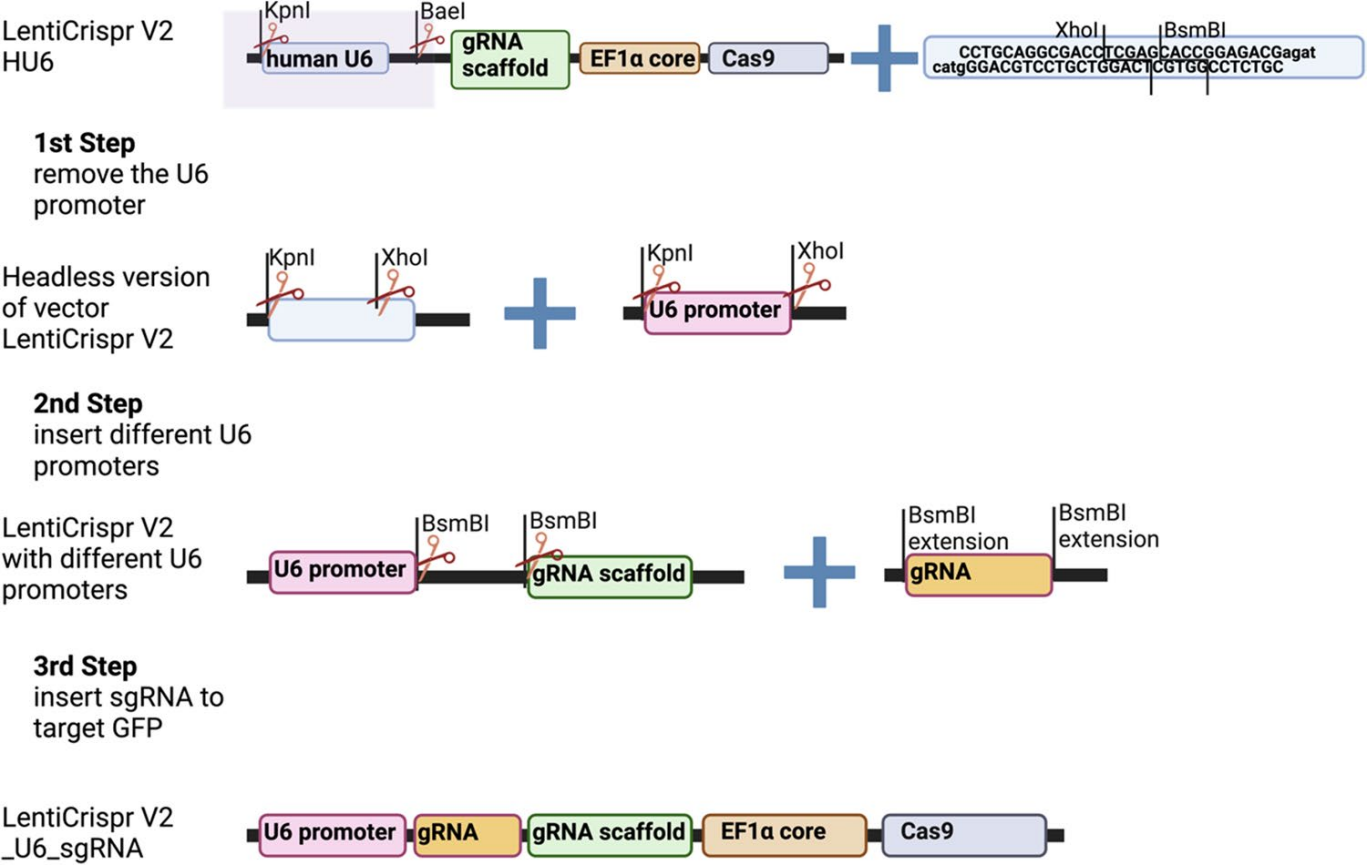
Construction of plasmids containing different U6 promoters and gRNA in three steps. In the first step, U6 less lentiCRISPR v2 plasmid was created by replacing the human U6 promoter with a 38 bp sequence containing *KpnI* and *BaeI* restriction sites. In the second step, different U6 promoters were inserted using the *KpnI* and *XhoI* restriction sites. And in the third step, LentiCrispr V2_different_U6_sgRNA plasmids were created by inserting gRNA using the *BsmBI* restriction site. Traditional cloning method was used for cloning in all the steps and Sanger sequencing was done to confirm all the sequences

To measure the activity of different U6 promoters, we inserted gRNA for GFP under different promoters (described in this section) and measured the expression of the GFP gRNA with qPCR (“Transfection of Plasmids for U6 Activity Assay” and “RNA Isolation, Reverse Transcription, and qPCR Analysis in Transfected Cells” sections). The lentiviral vector lentiCRISPR v2 from Feng Zhang’s lab (Addgene #52,961; Sanjana et al. 2014) was used as a starting point. Different promoters were cloned in place of the endogenous hU6 promoter in three steps: (1) remove the U6 promoter creating a “headless” version of the lentiCRISPR V2 vector, (2) insert our U6 promoters of interest, (3) insert sgRNA to target GFP (Fig. 1). Each of these steps is described in detail below.

To generate the headless lentiCRISPR vector, we digested lentiCRISPR v2 with *KpnI* and *BaeI* and replaced the U6 promoter with a 38bp sequence which included *XhoI* and *BsmBI* restriction sites to facilitate the insertion of other U6 promoters. The sequence which had *BaeI* and *KpnI* overhangs was made by annealing the oligos prHeadlessU6_fwd and prHeadlessU6_rev together (Table S2). The protocol was as follows: 1 µl of each oligo (100 µM; Invitrogen) was added to 7 µl of nuclease-free water. Then, 1 µl of T4 buffer (ThermoFisher) was added and the mixture was heated to 95 °C and cooled to room temperature at a 1% ramp rate. Then, the annealed product was diluted at 1:200 in elution buffer. After that, the lentiCRISPR v2 was digested using *KpnI* and *BaeI* (New England Biolabs), gel purified using a NucleoSpin® Gel and PCR Clean-up kit (AH-diagnostics), and ligated to the annealed U6 oligos using 50 ng backbone, 1 µl insert, and T4 DNA ligase (ThermoFisher) in a 1-h reaction at room temperature. Two microliters of ligation was transformed into One Shot™ Stbl3™ competent *E. coli* cells (ThermoFisher). The transformed *E. coli* cells were plated and single colonies were amplified in LB medium the following day. The recombinant plasmids were extracted by using the ZymoPURE Plasmid Kit (Zymo). Plasmids were verified via sanger sequencing (Eurofins Genomics) targeting the cloning junction (Table S2, primer-20 lenti insert u6, gRNA check).

Next, we inserted our U6 promoters into the headless U6 region to produce the modified U6 plasmids. For cloning, *KpnI* cut sites were included along with five extra base pairs in each forward primer designed, and *XhoI* cut sites were included after five extra base pairs in each reverse primer. sU6 promoters were amplified by using the genomic DNA of Atlantic salmon, and mU6 was amplified by using pSico (Addgene #11,578). For zU6, fU6, medU6, and tU6, promoters’ synthetic DNA was ordered from Integrated DNA Technologies (IDT). Platinum™ II Taq Hot-Start DNA Polymerase (Invitrogen) was used to clone the promoters. The protocol was as follows: 94 °C for 2 min, three cycles of 94 °C for 15 s, 60 °C for 15 s, and 68 °C for 15 s, then 32 cycles of 94 °C for 15 s, 58 °C for 15 s, and 68 °C for 15 s. Finally, the reaction was cooled to 4 °C. After confirming the product size on a 1% agarose gel, the PCR products were digested using *KpnI* and *XhoI* restriction enzymes and cleaned up using DNA Clean & Concentrator (Zymo). The headless version of the plasmid was digested using the same restriction enzymes, and gel purified according to previously mentioned protocol. Ligation of different promoters was done by using T4 DNA ligase (Thermo Scientific) in a 1:5 ratio (vector: insert) for 1 h at room temperature. The resulting vector was then transformed by using One Shot™ Stbl3™ competent *E. coli* cells. The correct insertions were confirmed as described earlier by using the same primer.

Lastly, to measure the U6 promoter activity, a gRNA targeting GFP (GGGCGAGGAGCTGTTCACCG) was inserted in the different U6 promoter plasmids using annealed oligo cloning. This gRNA will be able to target the GFP gene of GFP transgenic cell lines, which could be useful for future experiments. The forward (gfp_ko_fwd, primer-6) and reverse (gfp_ko_rev-7) oligonucleotide was designed with *BsmBI* cut sites in the overhang and annealed together according to the previously mentioned protocol. Plasmids were digested with the same restriction enzymes, gel purified, and the gRNA was ligated into the linearized plasmid. The vector was transformed into One Shot™ Stbl3™ *E. coli* cells, and purified plasmids were confirmed with Sanger sequencing using primer-20 (Table S2).

#### Transfection of Plasmids for U6 Activity Assay

The transfection protocol described by Gratacap et al. (2020) was followed, with some modifications. For the U6 activity assay, 2 µg plasmids were mixed with 10 µl of SHK-1 cells at 2*10^7^ cells/ml in Opti-MEM Reduced Serum Media Neon transfection system (Invitrogen) and Neon transfection 10 µl Kit (Invitrogen) was used to transfect the plasmids. The protocol used for the transfection was 1050 V, 30 ms, 2 pulses. Cells were kept in antibiotic-free culture media overnight. The next day media was replaced with fresh culture media described in 2.1 containing antibiotics.

#### RNA Isolation, Reverse Transcription, and qPCR Analysis in Transfected Cells

Seven days after electroporation, cells were collected, washed twice with PBS and the RNA was isolated using RNeasy Plus mini-Kit (Qiagen). TURBO DNA-free™ Kit (Invitrogen) was used to remove plasmid DNA. Seven hundred fifty nanograms of RNA was used in a 16 µl reaction. The reactions were incubated at 37 °C for 30 min, and later the DNaseI was deactivated by heating the mix at 75 °C for 10 min, then adding 2 µl of 50 mM EDTA. The integrity of the RNA was checked with the Agilent 4150 TapeStation System (Agilent) using RNA ScreenTape assay. Next, RNA was reverse transcribed using QuantiTect Reverse Transcription Kit (Qiagen), where 2 µl of DNA wipeout (supplied with kit) was added to 12 µl of previously DNaseI-treated RNA, incubated at 42°C for 5 min, and kept on ice until further use. A small volume from all of DNaseI-treated RNA samples was saved as a negative control for the qPCR. For the rest of the RNA, reverse transcription was done using random primer in a 20 µl reaction volume with 30 min of incubation at 42 °C. The cDNA obtained from the reverse transcription was utilized as the template for the qPCR reactions, which were conducted on a CFX96 Touch™ Real-Time PCR Detection System (Bio-Rad) with the use of SYBR select master mix (ThermoFisher). Each cycle of the PCR was 95 °C for 15 s and 63 °C for 1 min. Samples were run in triplicate and Cas9 was used as an internal control, because all the different U6 plasmids had the same EF1α core promoter driving Cas9 expression. Therefore, we assumed it would give the same level of expression. Primer efficiencies were determined using serial dilution of Human U6 plasmid with the help of Bio-Rad CFX Manager™ Software which uses the following equation: primer efficiency E = 10^(-1/slope). The GFP_KO_qPCR primer pair had 104.6% efficiency and Cas9_KO_qPCR primer pair had 97.4% efficiency. Primer sequences were checked against the salmon genome sequence using NCBI Primer-BLAST, and no binding sites were found. Melting curve analysis was performed after each qPCR run and analyzed with the Bio-Rad CFX Manager ™ Software. All PCR reactions had a single, sharp peak, indicating high primer specificity. Primer sequences are listed in Table 1. All reactions were conducted with three technical replicates and three biological replicates performed on different days. Relative expression of gRNA from each U6 promoter was calculated and compared to the expression of gRNA from hU6 promoter using the 2^−ΔΔCT^ method (Livak & Schmittgen 2001).

**Table 1:** Sequences of oligos and primers used in this study

Primer name	Sequence	Purpose
prHeadlessU6_fwd	CCTGCAGGCGACCTCGAGCACCGGAGACGAGAAT	Making Hybrid sequence (U6 assay)
prHeadlessU6_rev	CGTCTCCGGTGCTCGAGGTCGCCTGCAGGGTAC	Making Hybrid sequence (U6 assay)
mouseU6_F1_KpnI	CGATggtaccGATCCGACGCGCCATCTC	Cloning U6 promoter
mouseU6_R1_XhoI	AGATctcgagAATTACTTTACAGTTAGGGTGAGTT	Cloning U6 promoter
zebrafishU6_F1_KpnI	TACGggtaccCACCTCAACAAAAGCTCCTCG	Cloning U6 promoter
zebrafishU6_R1_XhoI	ACTActcgagTGGGAGTCTGGAGGACGG	Cloning U6 promoter
salU6_F1_KpnI	TGAGGTACCAGTGTACTTGCATATCACCCAGC	Cloning U6 promoter
salU6_R1_XhoI	TAGACTCGAGAATACAGACATaggagaggccctc	Cloning U6 promoter
medaka_U6_F1_KpnI	TACGggtaccGTGGGGATTGGTAAAGGTCC	Cloning U6 promoter
medaka_U6_R1_XhoI	ACTActcgagCGATGAGCCAAAGTCTCTGAG	Cloning U6 promoter
fugu_U6_F1_KpnI	TACGggtaccCACTGGATCCATCTGACACTAAG	Cloning U6 promoter
fugu_U6_R1_XhoI	ACTActcgagAACAGAGAGCAACATCTACGTGG	Cloning U6 promoter
tilapia_U6_F1_KpnI	TACGggtaccCTGAAGTATACTATGTGCCGAAT	Cloning U6 promoter
tilapia_U6_R1_XhoI	ACTActcgagCGACAGCTCCAAGGACCC	Cloning U6 promoter
GFP_KO_qPCR_F	GCGAGGAGCTGTTCACCG	qPCR (U6 Assay)
GFP_KO_qPCR_R	ACTCGGTGCCACTTTTTCAAG	qPCR (U6 Assay)
Cas9_KO_qPCR_F	ACCTATGCCCACCTGTTCG	qPCR (U6 Assay)
Cas9_KO_qPCR_R	AGGATTGTCTTGCCGGACTG	qPCR (U6 Assay)

### Polymerase Type II Promoters

#### Selection and Characterization of Pol II Promoter Candidates

The selection of candidate promoters was based on RNA-seq data from an Atlantic salmon tissue panel (Gillard et al. 2018). Highly expressed genes across different tissues were identified and the constitutive promoter candidates (Table S1 for NCBI accession number, File S1 for sequence) were picked from the upstream regions of these genes. The open chromatin regions for each candidate promoter were analyzed using ATAC-seq data from SalmoBase (https://salmobase.org/). The entire open chromatin region plus a few bp flanking was considered promoter candidate for analysis. The candidate promoters were sNUC3L, sETF, sXRCC1L, and three putative heat shock promoters (sHSP7C, sHSP70-3, and sHSP8) (Table S1 for NCBI accession number, File S1 for sequence). Analysis of ATAC-seq data showed there were two peaks and RNA-seq data showed gene expression on both sides, suggesting that sNUC3L and sETF could be bidirectional. In a preliminary luciferase assay, we measured the activity of the candidate promoters mentioned above in addition to the sNUC3L and sETF in reverse direction (sNUC3L-R, sETF-R). In the final experiment, the sNUC3L-R, sETF (both directions), sXRCC1L, sHSP7C, and sHSP70-3 were excluded due to low activity in the preliminary experiment and endogenous sEF1α promoter was added along with SV40 for comparison with the hEF1α core which would give a broad perspective of promoter activity.

**Fig. 2:**
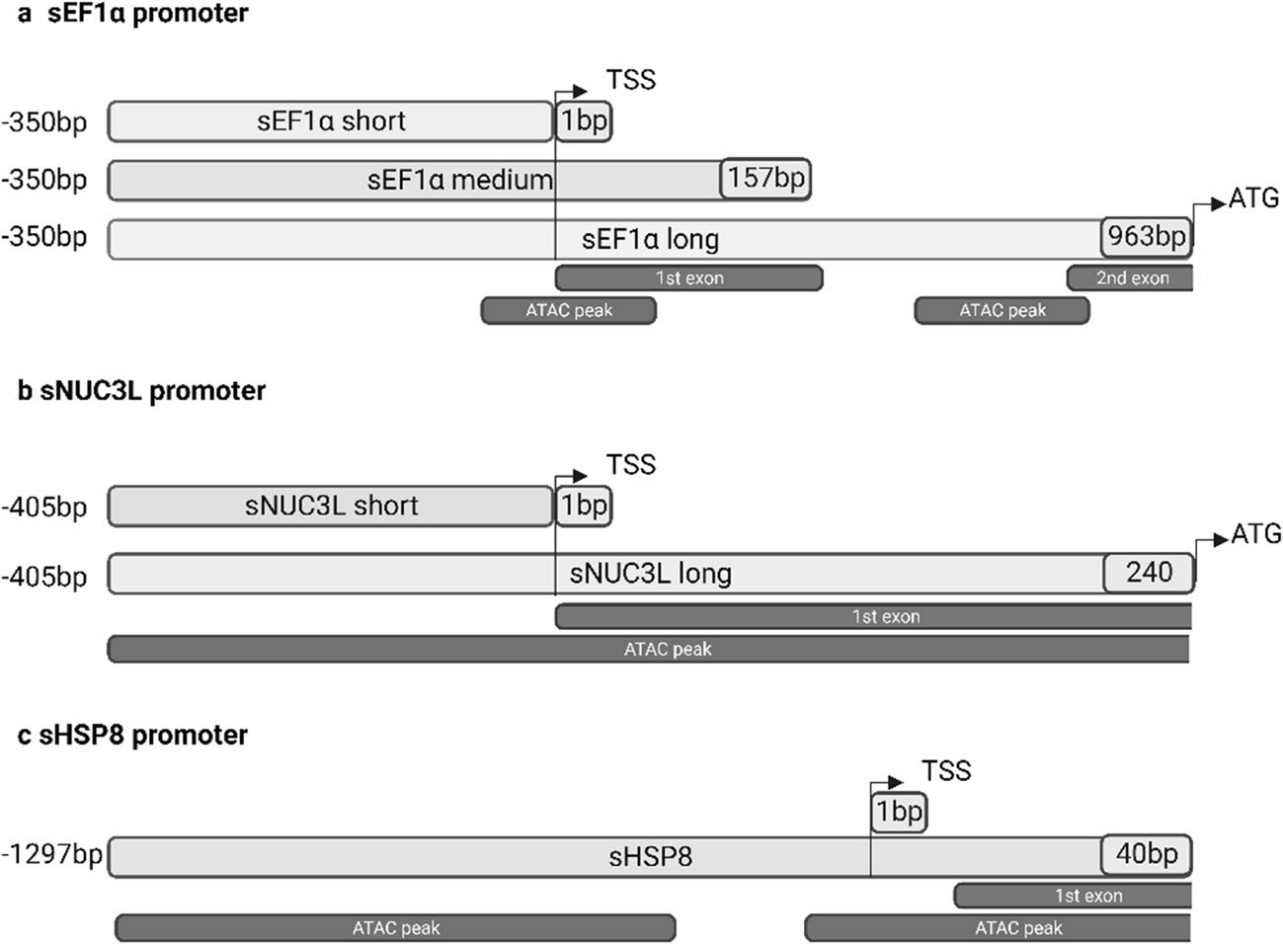
Endogenous promoter candidates: sEF1α (A), sNUC3L (B), and sHSP8 (C). Negative base pairs (bp) indicate the bp upstream of the transcription start site (TSS). Approximate positions of ATAC peaks and exon locations are denoted below the sequences

To further characterize the candidate promoters, different lengths of the sEF1α and sNUC3L promoters were selected based on the ATAC-seq data. The sEF1α promoter candidate had two ATAC peaks before the coding sequence (Fig. 2A) with the first peak starting 81 bp upstream of the TSS (transcription start site) and extending 205 bp downstream of the TSS, including a portion of the first exon. The second peak started from 567 to 723 bp downstream of the TSS, including a portion of the second exon but not the start codon. Based on the ATAC-seq data, we speculate that the sEF1α promoter is spanning from − 81 bp upstream of TSS all the way to the start codon 723 bp downstream of TSS. To find out if the regions downstream of the TSS are important for expression, three lengths were selected for further experiments: sEF1α, sEF1α medium, and sEF1α short. The sEF1α short promoter extended up to the first exon (excluding the first exon), the sEF1α medium promoter included the first exon, and the sEF1α promoter extended to a portion of the second exon (excluding the start codon).

The sNUC3L promoter candidate had one ATAC peak starting from 405 bp upstream of the TSS and extending to 535 bp downstream of the TSS, including the first exon and a portion of the coding region (Fig. 2B). Two lengths of the sNUC3L promoter were used in further analysis and experiments: sNUC3L short and sNUC3L long. The sNUC3L short promoter extended up to the first exon (excluding the exon), and the sNUC3L long promoter extended up to the first codon (excluding the ATG). For sHSP8 promoter, a 1337 bp fragment was identified, with one ATAC peak 1297 bp upstream of the TSS and one ATAC peak starting before the TSS and extending 40 bp into the untranslated region (Fig. 2C).

#### Sequence Analysis

NCBI and SalmoBase were used to investigate the associated gene sequences, exon intron regions, and coding sequences. The regulatory motifs of the promoter sequences were detected by using FIMO analysis (Grant et al. 2011) where candidate promoters were scanned against the JASPAR2022 CORE vertebrates’ non-redundant motif database (https://jaspar.genereg.net/downloads/). The potential motif matches for pol II promoters were filtered out by setting the *p*-value to *p* < 1E^−6^. A possible PRDM1 motif which was present in sEF1α was investigated to test the significance of the suggested motif for promoter activity. A random single bp mutation was introduced by PCR in the location of the PRDM1 motif of sEF1α promoter and named sEF1α ΔPRDM1. The mutation in PRDM1 was introduced unintentionally by the Taq polymerase used in cloning of the sEF1α promoter (see details of the PCR reaction in the next section). In short, we amplified the sEF1α promoter and ligated the sequence into vectors and these vectors were used to transform *E. coli*. Vectors from 20 *E. coli* colonies were sequenced and one vector contained a mutation in the PRDM1 motif, where the 10th base of PRDM1 motif was changed from a T to a C. The motif comparison tool Tomtom was used to compare binding properties of the mutated sequence to known binding sites for PRDM1 (Gupta et al. 2007).

#### Construction of Luciferase Reporter Plasmid for the Assay

To clone the promoters into the luciferase vector, pGL4.10 (luc2; Promega, USA), *KpnI* and *XhoI* restriction enzymes were used to digest the plasmid. Restriction enzyme cutting sites were added to the different promoter amplicons by including primer overhangs (Table S2). All endogenous promoters were amplified from salmon genomic DNA. SV40 and hEF1α core promoters were amplified from lentiCRISPR v2 (Sanjana et al. 2014), and PGK promoter was amplified from pKLV2-U6gRNA5(BbsI)-PGKpuro2ABFP-W (Addgene #67,974). The PCR protocol from the “Production of Vectors with Different U6 Promoters for Evaluating gRNA Expression Efficiency” section was followed, except for a bit longer extension time (15 s/kb). The PCR products were digested with *KpnI* and *XhoI*, and purification and ligation were done according to the previously described protocol. After ligation, DH5α Competent Cells with the resulting vector plated and cultured as described above. After plasmid isolation, constructs were verified by Sanger sequencing in the cloning junction by using sequencing primer (Table S2: Primer 47, insert check_pgl4.10).

#### Transfection of SHK-1 Cells with Promoter Constructs

For the luciferase assay, 1.9 µg promoter plasmids and 100 ng of reporter plasmid (Renilla luciferase) were mixed with 10 µl of SHK-1 cells at 2 × 10^7^ cells/ml in Opti-MEM Reduced Serum Media (Gibco) and then the transfection was done by electroporation as described previously (“Transfection of Plasmids for U6 Activity Assay” section).

#### Heat Shock Treatment for HSP Promoters

To determine the effect of temperature on the expression of luciferase controlled by heat shock promoters, a separate luciferase assay was performed. The cells were subjected to different temperature conditions after the transfection to determine the effect on luciferase expression. The PGK-luciferase plasmid showed stable expression among the groups and was used as a control in the different temperature groups. For this assay, in the first experiment, cells were kept at 15 °C for 2 h after transfection and then separated into groups that were transferred to 20 °C, 24 °C, and 26 °C for 2 h before the luciferase assay. In the second experiment, wild cells were kept at 12 °C for a week before transfection and transferred in separate groups to 9 °C, 12 °C (control), and 20 °C for 2 and 6 h before the luciferase assay. In a third experiment, sHSP8 transfected and wild cells were kept at 8 °C for a week before transfection and transferred in separate groups to 8 °C (control), 12 °C, 15 °C, and 20 °C for 6 h before the luciferase assay.

#### Luciferase Assay

The firefly and Renilla luciferase activities were measured using a Dual-Glo Luciferase Assay system (Promega, USA) on a Synergy H1 Hybrid reader (Biotek) after 48 h of transfection. To each well, 85 µL of DMEM was added replacing the L15 media. Then, an equal volume of Dual-Glo luciferase reagent was added, and the firefly luciferase activities were measured. The Renilla luciferase activities were measured after adding 85 µl of Dual-Glo Stop & Glo reagent to each well. The promoter activity for each plasmid construct was calculated by dividing the average firefly luciferase activity by the average Renilla luciferase activity, which was obtained by measuring three technical replicates. Three independent experiments were performed in different cell passages, with each plasmid construct transfected in triplicate.

$$Promoter activity= \frac{Average firefly luciferase activities}{Average renila luciferase activities}$$

### Statistical Analysis

Data were analyzed with GraphPad Prism 9.4.1 using a one-way variance analysis followed by the Tukey multiple comparison test at *p* < 0.05. All values are expressed as mean ± SE.

## Results

### In SilicoAnalysis of the Promoter’s Regulatory Regions

#### Pol III Promoters

**Fig. 3:**
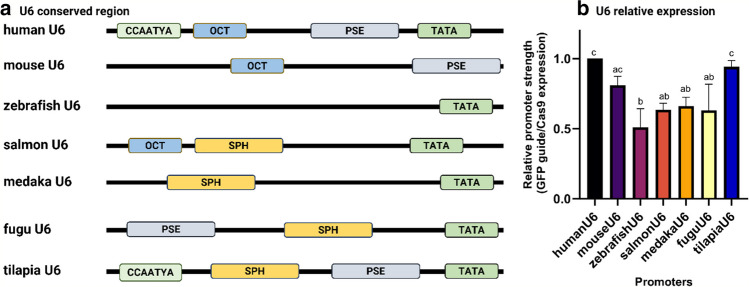
Characterization and relative expression of different U6 promoters. (A) Characterization of different U6 promoters. The FIMO (Find Individual Motif Occurrences) was used to scan for highly conserved regions in the sequences. Consensus sequences for CCAATYA, Octamer (OCT), Proximal Sequence Element (PSE), SPH, and TATA box were obtained from already published paper and scanned against U6 promoter in FIMO. The potential motif matches were filtered by setting the *p*-value to *p* < 0.001. (B) Relative promoter strength of different U6 promoters measured by qPCR (*n* = 3). Cas9 was used as an internal normalization control. The relative expression of each U6 promoter was measured and normalized by the 2^−ΔΔCt^ method. Data are shown as the mean ± S.E and different letters above each column indicate statistically significant differences (*p* < 0.05) determined by one-way ANOVA followed by Tukey’s multiple comparison test

**Fig. 4:**
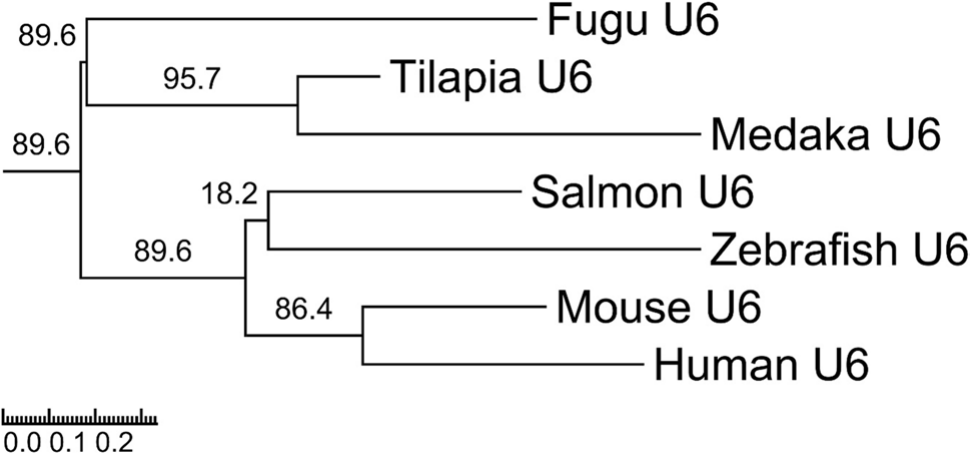
Phylogenetic tree of different U6 promoters. Alignment and phylogenetic reconstructions were performed utilizing the “build” function of ETE3 version 3.1.2 as incorporated within the GenomeNet platform (https://www.genome.jp/tools/ete/) and with the full sequence of each promoter as input

The motif analysis revealed that all 65 salmon U6 promoters contained the TATA box (see Supplementary File S2 for all sequences used in the analysis). Sixty-two other salmon U6 promoters had only one additional motif, which was predominantly the CCAATYA motif. Only two promoters with two additional key U6 motifs were identified. The LOC123731961 promoter contained TATA, CCAATYA, and PSE motifs, while the LOC123740086 promoter (our candidate salmon U6 promoter) contained TATA, OCT, and SPH motifs (Fig. 3A). We also found that hU6 had the TATA, CCAATYA, OCT, and PSE motifs, tU6 had TATA, CCAATYA, SPH, and PSE motifs, and fU6 had TATA, PSE, and SPH motifs. Interestingly, we could only identify one motif in zU6, the TATA motif; however, the phylogenetic analysis of the U6 promoters suggests that sU6 is most closely related to zU6 (see Fig. 4).

#### Pol II Promoters

**Fig. 5:**
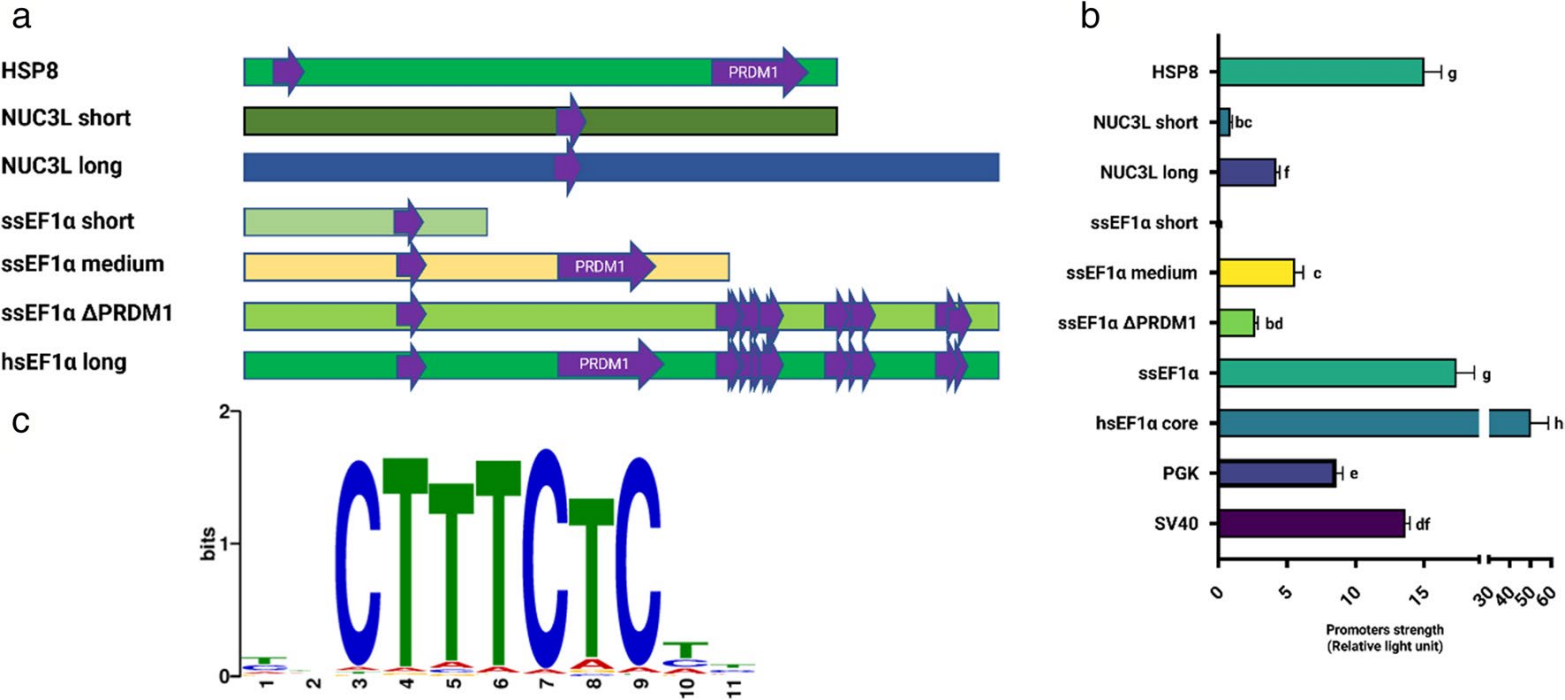
Characterization of promoters and gene expression. (A) Potential transcription factor binding sites of different pol II promoters and their approximate locations are indicated in the purple arrow box. The FIMO (Find Individual Motif Occurrences) was used to identify the motifs. JASPAR2022 CORE vertebrates’ non-redundant database was used against targeted promoters. The potential motif matches for pol II promoters were filtered out by setting the *p*-value to *p* < 1E − 6. (B) Promoters’ expression efficiency. Each promoter activity was calculated by dividing the average luciferase activities of three technical replicates of firefly by Renilla luciferase. The experiment was conducted with three biological replicates to demonstrate the reproducibility of the results. The figure displays data from one biological replicate, while the other two replicates are presented in Supplementary Fig. S2. Data were analyzed with GraphPad Prism 9.4.1 using a one-way variance analysis followed by the Tukey multiple comparison test. All values are expressed as mean ± SE and different letters signify statistical differences (*p* < 0.05). (C) PRDM1 motif logo built by the motif comparison tool Tomtom in the MEME suite showed that T bp has a higher occurrence rate in the 10th bp region compared to C bp

We identified 3 putative heat shock promoters (HSP70-3, HSP7C, sHSP8), 3 putative constitutive salmon promoters (sNUC3L, ETF, XRCC1l), and the sEF1α promoter based on RNA-seq data from 15 tissues and ATAC-seq data from the liver. Motif analysis of these promoters showed that the sHSP8 promoter contains putative binding sites for BCL6 and PRDM1. HSP7C contains AT2G40260 and two Fox family transcription factors. sNUC3L promoter contains a putative binding site for CTCF. ETF contains binding sites for several Fox family and other transcription factors. The sEF1α promoter contains putative binding sites for AGL27, AT1G19040, DOF3.6, DUX, NAC071, PRDM1, TCX6, and four WRKY family transcription factors (Fig. 5A for approximate location of different promoters). Interestingly, we could not identify any TATA box and CAAT box in the promoters sHSP8, sNUC3L, and sEF1α. All motif sequences can be found in Table S3.

A single base pair mutation (T to C) in the sEF1α promoter resulted in the loss of the PRDM1 binding site. We used the motif comparison tool Tomtom to compare the mutated sequence to known binding sites for PRDM1 and confirmed that the T base pair is more commonly observed in PRDM1 binding sites than the C base pair (Fig. 5C).

### Activity of U6 Promoters in SHK-1 Cells

To estimate the relative promoter strength of different U6 promoters, we compared the RNA level of a gRNA targeting GFP under different U6-promoters in SHK-1 cells. The results were compared to the RNA levels from the human U6 promoter, which was set to 1. Because the U6 promoters were inserted in lentiCRISPR v2 plasmids which also contain Cas9 under the EF1core promoter, Cas9 mRNA was used to normalize the results for variation in transfection efficiency. qPCR of the GFP gRNA showed that human U6 promoter (hU6) and tilapia U6 promoter (tU6) gave the highest level of gRNA expression (Fig. 3B), followed by mouse U6 promoter (mU6) (Fig. 3B). Interestingly, salmon U6 promoter (sU6), medaka U6 promoter (medU6), and fugu U6 promoter (fU6) showed similar expression levels, but lower than that of mU6. The lowest level of expression was observed in the zebrafish U6 (zU6) promoter.

### Activity of Polymerase II Promoters in SHK-1 Cells

Pol II promoter activity was evaluated by luciferase assay. All promoters were cloned into the pGL4.10 vector upstream of firefly luciferase and co-transfected with Renilla luciferase into SHK-1 cells. We found that the CMV promoter expressed firefly luciferase at the highest level, with a 485-fold induction compared to Renilla luciferase, while the PGK, sNUC3L, and sHSP8 promoters showed promising expression with 2.5-, 1-, and threefold induction respectively. sNUC3L-R, ETF-R, XRCC1L, HSP7C, and HSP703 had very low expression ( Supplementary Fig. S1) and were left out of future experiments. Future experiments also excluded CMV due to its extremely high activity.

Luciferase assays of the final cohort of promoters showed that hEF1α core gave the highest expression with 2–threefold more signal than the next highest expressing promoter, sEF1α and sHSP8 (Fig. 5b; Fig. S2). SV40 and PGK had significantly higher expression than other studied promoters but had lower luciferase expression than sHSP8, sEF1α, and hEF1α core. To further characterize the activity of selected candidate promoters, different lengths of the sEF1α and sNUC3L were tested by luciferase assay. Our results showed that the sNUC3L long promoter has approximately fourfold higher expression than the ssNUC3L short promoter. Among the sEF1α promoters, the sEF1α promoter demonstrated significantly higher expression than the sEF1α medium promoter. However, the sEF1α short promoter did not show any expression.

In order to investigate the effect of the PRDM1 binding motif on the activity of the sEF1α promoter, we knocked out the PRDM1 motif by single base pair mutation (sEF1α ΔPRDM1). Interestingly, there was a significant difference in expression efficiency between sEF1α and sEF1α ΔPRDM1. sEF1α has about eightfold higher expression efficiency than the sEF1α ΔPRDM1. These findings suggest that the PRDM1 binding motif in the sEF1α promoter greatly enhances its activity.

### Heat Shock Treatment for the HSP Promoter

To investigate the effect of temperature on the heat shock promoter activity, a separate luciferase assay was performed using only the HSP promoters. In the heat shock experiment, different temperature treatments did not show any significant increase in luciferase expression for the HSP promoters. The cells were subjected to various heat shock strategies, including heat shock at different temperatures (24 °C, 26 °C) for 2 or 6 h, and cold acclimatization prior to heat shock (8 °C, 12 °C, 15 °C), but no significant differences in luciferase expression were observed between the treatments and controls (Supplementary Fig. S3).

## Discussion

Appropriate expression of Cas9 and gRNA is crucial for the success of gene editing using vectors or lentivirus transduction. For U6 promoters that drive the expression of gRNAs, we saw that the activity of human and tilapia U6 promoters in SHK-1 cells was highest among those we tested (Fig. 3B) and should be considered for use in future experiments in Atlantic salmon cells. The U6 promoter from the more phylogenetically related zebrafish had the lowest activity in our experiments, with about the half of the activity of the human U6 promoter (Fig. 3B). Mouse, medaka, and fugu U6 promoters had together with the endogenous promotor from salmon a relatively intermediary activity, suggesting that phylogeny is not necessarily a factor in how well a promoter work. Some previous studies in fish have shown similar results, with human U6 having higher activity than other U6 promoters in some fish cells (Mäkinen et al. 2006; Roelz et al. 2010). However, in a tilapia cell line, human U6 showed no activity (Hamar & Kültz 2021), while the endogenous tilapia promoter had very high activity. The discrepancy in the results might be explained by different assays. However, it might show the importance of testing the activity of the promoters in each specific cell line.

Promoter activity is heavily dependent on the presence of transcription factor binding sites in the promoter sequence. Promoter regions of snRNA genes in vertebrates include a TATA box, a PSE, and a DSE with submotifs SPH and OCT, which may exist together or separately (Schaub et al. 1997; Schramm & Hernandez 2002). Indeed, in silico analysis of our promoters identified these binding sites in some of our studied U6 promoters (Fig. 3A). Interestingly, the two U6 promoters showing the highest activity in SHK-1 cells in our experiments, human and tilapia U6 promoters, both contained a CCAATYA motif which was absent in all other promoters tested. This motif is recognized by a family of transcription factors called CCAAT-enhancer binding proteins (C/EBPs), which are crucial for the regulation of various biological processes, such as cell differentiation, immune response, and metabolism (Landschulz et al. 1988; Osada et al. 1996). Mantovani (1998) reported that the CCAAT box is a critical *cis* element for the regulation of a large number of eukaryotic genes (Mantovani 1998). Different experiments using site-directed mutagenesis have demonstrated that the presence of the CCAAT box can significantly enhance the activity of various promoters (Bucher 1990). These findings suggest that the presence of the CCAATYA motif in the human and tilapia U6 promoters may be an important factor contributing to their higher expression levels.

For the pol II promoters, we found that the heterologous promoter CMV activity was very high in SHK-1 cells, with activity almost tenfold of the second highest promoter, human EF1a core. This could possibly be too high activity for driving Cas9 expression, since several papers report that too high Cas9 level could be toxic for the cells (Ferreira et al. 2021; Li et al. 2018). Several studies have found that heterologous promoters can be stronger than endogenous promoters in certain situations, while the opposite can be true in other cases. In Japanese flounder (*Paralichthys olivaceus*) cells, the heterologous promoter CMV activity was stronger than the endogenous promoter beta-actin (B. Wang et al. 2018). Conversely, in fathead minnow (*Pimephales promelas*) and tilapia (*Oreochromis mossambicus*) cells, endogenous promoters showed stronger expression than the CMV promoter (Hamar & Kültz 2021; Ruiz et al. 2008). hEF1α promoter has been shown to be a strong promoter in various mammalian cell lines (Kim et al. 2002), and here we show that this is also the case in SHK-1 cells. We tested three different lengths of the sEF1α promoter sequence (Fig. 5). sEF1α contains the first intron and part of second intron of the sEF1α gene and displayed the highest activity followed by the sEF1α medium which has the first exon of sEF1α gene and the sEF1α short promoter which had no activity. Similarly, Kim et al. (2002) demonstrated that insertion of the first intron of EF1α in front of the murine CMV promoter led to increased gene expression levels in several cell lines (Kim et al. 2002). Previous research has also suggested that the first intron enhancer elements are necessary for potent β-actin promoter activity in other species (Kawamoto et al. 1988; Liu et al. 1990). The presence of several transcription factor binding sites in untranslated region might be the reason of higher activity of sEF1α and sEF1α promoters and sNUC3L long promoters. The 5′ UTR can influence mRNA stability, translation efficiency, and subcellular localization through various mechanisms, such as presence of additional transcription factors, secondary structures, RNA-binding protein interactions, or internal ribosome entry sites (IRESs) (Jackson et al. 2010, 2010; Kozak 2005).

The promoter sequences of pol II promoters sHSP8, sNUC3L, and sEF1α were analyzed in silico for the presence of transcription factor binding sites, which regulate gene expression by recruiting RNA polymerase for transcription. TATA box and CAAT-box, which are common in eukaryotic promoters, were not predicted in the analyzed sequences. Only 5–7% of eukaryotic promoters have a TATA box, while the CAAT-box is more common but still not present in the majority of promoters (Roy & Singer 2015; Xiao-hui 2008). Interestingly, the PRDM1 transcription factor was predicted to be present in both sHSP8 and sEF1α promoter sequences which displayed similar expression levels. Interestingly, a study on *Pilchard orthomyxovirus* (POMV) infection in Atlantic salmon (*Salmo salar*) suggested that PRDM1 plays a central role as a key regulatory factor during the infection with POMV (Samsing et al. 2020). We found that deletion of the sEF1α PRDM1 motif severely reduced promoter activity. Further, ATAC-seq of salmon liver tissue shows that the PRDM1 binding site is located in the open chromatin region of both sHSP8 and sEF1α promoters. Previous research has shown that zinc finger domains in PRDM1 mediate DNA binding, nuclear import, and recruitment of histone-modifying enzymes (Bikoff et al. 2009; Morgan et al. 2012; Varshney et al. 2021). One potential mechanism by which PRDM1 may enhance promoters’ activity is through the modulation of chromatin structure, possibly by performing histone modifications, resulting in an open chromatin structure, and increased transcriptional activity. Alternatively, PRDM1 may interact with other regulatory factors to increase the expression.

Finally, even if we often think of promoters as the sequence directly upstream of the transcription start site, our results from experiments with different length of two promoters (sNUC3L and sEF1α) indicate that the sequence all the way downstream to the start codon (ATG) could possibly be a part of the promoter. However, including this sequence will increase the size of the synthetic promoter, something we want to avoid due to less efficient transfection of large plasmids. To determine the exact size of the minimal promoter sequence, we recommend conducting further experiments involving the deletion of portions of the sequence, starting from the ATG, and moving backward. Also, removing parts of the other side (upstream of TSS) of the promoter or parts within the promoter should be tested. This will allow for a more precise identification of the minimal promoter region.

In conclusion, our study provides valuable insight into the performance of different promoters in Atlantic salmon cells and will facilitate future transgenic research, gene editing, and CRISPR screening.

## Supplementary Information

Below is the link to the electronic supplementary material.

Supplementary file1 (TIF 960 KB)

Supplementary file2 (TIF 1403 KB)

Supplementary file3 (TIF 844 KB)

Supplementary file4 (DOCX 51 KB)

Supplementary file5 (DOCX 20 KB)

Supplementary file6 (DOCX 16 KB)

## Data Availability

No datasets were generated or analysed during the current study.

## References

[CR1] Bikoff EK, Morgan MA, Robertson EJ (2009) An expanding job description for Blimp-1/PRDM1. Curr Opin Genet Dev 19:379–385 19592232 10.1016/j.gde.2009.05.005

[CR2] Blix TB, Dalmo RA, Wargelius A, Myhr AI (2021) Genome editing on finfish: current status and implications for sustainability. Rev Aquac 13:2344–2363

[CR3] Boonanuntanasarn S, Panyim S, Yoshizaki G (2009) Usage of putative zebrafish U6 promoters to express shRNA in Nile tilapia and shrimp cell extracts. Transgenic Res 18:323–325 19225901 10.1007/s11248-009-9249-0

[CR4] Boyd CE, D’Abramo LR, Glencross BD, Huyben DC, Juarez LM, Lockwood GS, McNevin AA, Tacon AGJ, Teletchea F, Tomasso JR, Tucker CS, Valenti WC (2020) Achieving sustainable aquaculture: historical and current perspectives and future needs and challenges. J World Aquac Soc 51:578–633

[CR5] Bucher P (1990) Weight matrix descriptions of four eukaryotic RNA polymerase II promoter elements derived from 502 unrelated promoter sequences. J Mol Biol 212:563–578 2329577 10.1016/0022-2836(90)90223-9

[CR6] Dahlberg JE, Lund E (1988) The genes and transcription of the major small nuclear RNAs. In M. L. Birnstiel (ed), Structure and function of major and minor small nuclear ribonucleoprotein particles (pp. 38–70). Springer. 10.1007/978-3-642-73020-7_2

[CR7] Dannevig BH, Brudeseth BE, Gjøen T, Rode M, Wergeland HI, Evensen Ø, McL Press C (1997) Characterisation of a long-term cell line (SHK-1) developed from the head kidney of Atlantic salmon (Salmo salar L.). Fish & Shellfish Immun 7:213–226

[CR8] Datsomor AK, Wilberg R, Torgersen JS, Sandve SR, Harvey TN (2023) Efficient transfection of Atlantic salmon primary hepatocyte cells for functional assays and gene editing. G3 13:039. 10.1093/g3journal/jkad039 10.1093/g3journal/jkad039 PMC10085798 36786483

[CR9] Didychuk AL, Butcher SE, Brow DA (2018) The life of U6 small nuclear RNA, from cradle to grave. RNA 24:437–460 29367453 10.1261/rna.065136.117 PMC5855946

[CR10] Duvoisin R, Ayuk MA, Rinaldi G, Suttiprapa S, Mann VH, Lee CM, Harris N, Brindley PJ (2012) Human U6 promoter drives stronger shRNA activity than its schistosome orthologue in *Schistosoma mansoni* and human fibrosarcoma cells. Transgenic Res 21:511–521 21953124 10.1007/s11248-011-9548-0 PMC3271131

[CR11] Escobar-Aguirre S, Arancibia D, Escorza A, Bravo C, Andrés ME, Zamorano P, Martínez V (2019) Development of a bicistronic vector for the expression of a CRISPR/Cas9-mCherry system in fish cell lines. Cells 8:75. 10.3390/cells8010075 30669572 10.3390/cells8010075 PMC6357165

[CR12] Ferreira FJ, Carvalho L, Logarinho E, Bessa J (2021) Foxm1 modulates cell non-autonomous response in zebrafish skeletal muscle homeostasis. Cells 10:1241. 10.3390/cells10051241 34070077 10.3390/cells10051241 PMC8158134

[CR13] Fourrier MCS, Munro ES, Fryer RJ, Hastings TS (2007) Long term monitoring of the virus susceptibility of five established fish cell lines. Bull Eur Ass Fish Pathol 27:192

[CR14] Gelinas R, Endlich B, Pfeiffer C, Yagi M, Stamatoyannopoulos G (1985) G to A substitution in the distal CCAAT box of the Aγ-globin gene in Greek hereditary persistence of fetal haemoglobin. Nature 313:323–325 2578619 10.1038/313323a0

[CR15] Geraghty RJ, Capes-Davis A, Davis JM, Downward J, Freshney RI, Knezevic I, Lovell-Badge R, Masters JRW, Meredith J, Stacey GN, Thraves P, Vias M (2014) Guidelines for the use of cell lines in biomedical research. Br J Cancer 111:1021–1046 25117809 10.1038/bjc.2014.166 PMC4453835

[CR16] Gervais O, Barria A, Papadopoulou A, Gratacap RL, Hillestad B, Tinch AE, Martin SAM, Robledo D, Houston RD (2021) Exploring genetic resistance to infectious salmon anaemia virus in Atlantic salmon by genome-wide association and RNA sequencing. BMC Genom 22:345. 10.1186/s12864-021-07671-6 10.1186/s12864-021-07671-6 PMC8117317 33985436

[CR17] Gillard G, Harvey TN, Gjuvsland A, Jin Y, Thomassen M, Lien S, Leaver M, Torgersen JS, Hvidsten TR, Vik JO, Sandve SR (2018) Life-stage-associated remodelling of lipid metabolism regulation in Atlantic salmon. Mol Ecol 27:1200–1213 29431879 10.1111/mec.14533

[CR18] Grant CE, Bailey TL, Noble WS (2011) FIMO: Scanning for occurrences of a given motif. Bioinform 27:1017–1018 10.1093/bioinformatics/btr064 PMC3065696 21330290

[CR19] Gratacap RL, Jin YH, Mantsopoulou M, Houston RD (2020) Efficient genome editing in multiple Salmonid cell lines using ribonucleoprotein complexes. Mar Biotechnol 22:717–724 10.1007/s10126-020-09995-y PMC7520412 32946000

[CR20] Gupta S, Stamatoyannopoulos JA, Bailey TL, Noble WS (2007) Quantifying similarity between motifs. Genome Biol 8:R24. 10.1186/gb-2007-8-2-r24 17324271 10.1186/gb-2007-8-2-r24 PMC1852410

[CR21] Hamar J, Kültz D (2021) An efficient vector-based CRISPR/Cas9 system in an *Oreochromis mossambicus* cell line using endogenous promoters. Sci Rep 11:7854. 10.1038/s41598-021-87068-3 33846462 10.1038/s41598-021-87068-3 PMC8041756

[CR22] Harvey AC, Solberg MF, Troianou E, Carvalho GR, Taylor MI, Creer S, Dyrhovden L, Matre IH, Glover KA (2016) Plasticity in growth of farmed and wild Atlantic salmon: is the increased growth rate of farmed salmon caused by evolutionary adaptations to the commercial diet? BMC Evol Biol 16:264. 10.1186/s12862-016-0841-7 27905882 10.1186/s12862-016-0841-7 PMC5134087

[CR23] Huerta-Cepas J, Serra F, Bork P (2016) ETE 3: reconstruction, analysis, and visualization of phylogenomic data. Mol Biol Evol 33:1635–1638 26921390 10.1093/molbev/msw046 PMC4868116

[CR24] Jackson RJ, Hellen CUT, Pestova TV (2010) The mechanism of eukaryotic translation initiation and principles of its regulation. Nat Rev Mol Cell Biol 11:113–127 20094052 10.1038/nrm2838 PMC4461372

[CR25] Jinek M, Chylinski K, Fonfara I, Hauer M, Doudna JA, Charpentier E (2012) A programmable dual-RNA–guided DNA endonuclease in adaptive bacterial immunity. Science 337:816–821 22745249 10.1126/science.1225829 PMC6286148

[CR26] Kawamoto T, Makino K, Niwa H, Sugiyama H, Kimura S, Amemura M, Nakata A, Kakunaga T (1988) Identification of the human beta-actin enhancer and its binding factor. Mol Cell Biol 8:267–272 3422098 10.1128/mcb.8.1.267-272.1988 PMC363114

[CR27] Kim S-Y, Lee J-H, Shin H-S, Kang H-J, Kim Y-S (2002) The human elongation factor 1 alpha (EF-1α) first intron highly enhances expression of foreign genes from the murine cytomegalovirus promoter. J Biotechnol 93:183–187 11738725 10.1016/s0168-1656(01)00388-1

[CR28] Kozak M (2005) Regulation of translation via mRNA structure in prokaryotes and eukaryotes. Gene 361:13–37 16213112 10.1016/j.gene.2005.06.037

[CR29] Landschulz WH, Johnson PF, McKnight SL (1988) The leucine zipper: a hypothetical structure common to a new class of DNA binding proteins. Science 240:1759–1764 3289117 10.1126/science.3289117

[CR30] Lhorente JP, Gallardo JA, Villanueva B, Carabaño MJ, Neira R (2014) Disease resistance in Atlantic salmon (*Salmo salar*): coinfection of the intracellular bacterial pathogen *Piscirickettsia salmonis* and the sea louse *Caligus rogercresseyi*. PLoS ONE 9:e95397. 10.1371/journal.pone.0095397 24736323 10.1371/journal.pone.0095397 PMC3988197

[CR31] Li C, Psatha N, Gil S, Wang H, Papayannopoulou T, Lieber A (2018) HDAd5/35++ adenovirus vector expressing anti-CRISPR peptides decreases CRISPR/Cas9 toxicity in human hematopoietic stem cells. Mol Ther Methods Clin Dev 9:390–401 30038942 10.1016/j.omtm.2018.04.008 PMC6054697

[CR32] Lifton RP, Goldberg ML, Karp RW, Hogness DS (1978) The organization of the histone genes in *Drosophila melanogaster*: functional and evolutionary implications. Cold Spring Harb Symp Quant Biol 42:1047–1051 98262 10.1101/sqb.1978.042.01.105

[CR33] Liu ZJ, Moav B, Faras AJ, Guise KS, Kapuscinski AR, Hackett PB (1990) Functional analysis of elements affecting expression of the beta-actin gene of carp. Mol Cell Biol 10:3432–3440 2355913 10.1128/mcb.10.7.3432-3440.1990 PMC360779

[CR34] Livak KJ, Schmittgen TD (2001) Analysis of relative gene expression data using real-time quantitative PCR and the 2(−ΔΔC(T)) method. Methods 25:402–408 11846609 10.1006/meth.2001.1262

[CR35] Mäkinen PI, Koponen JK, Kärkkäinen A-M, Malm TM, Pulkkinen KH, Koistinaho J, Turunen MP, Ylä-Herttuala S (2006) Stable RNA interference: comparison of U6 and H1 promoters in endothelial cells and in mouse brain. J Gene Med 8:433–441 16389634 10.1002/jgm.860

[CR36] Mantovani R (1998) A survey of 178 NF-Y binding CCAAT boxes. Nucleic Acids Res 26:1135–1143 9469818 10.1093/nar/26.5.1135 PMC147377

[CR37] Morgan MAJ, Mould AW, Li L, Robertson EJ, Bikoff EK (2012) Alternative splicing regulates Prdm1/Blimp-1 DNA binding activities and corepressor interactions. Mol Cell Biol 32:3403–3413 22733990 10.1128/MCB.00174-12 PMC3422002

[CR38] Osada S, Yamamoto H, Nishihara T, Imagawa M (1996) DNA Binding specificity of the CCAAT/enhancer-binding protein transcription factor family. J Biol Chem 271:3891–3896 8632009 10.1074/jbc.271.7.3891

[CR39] Pavelin J, Jin YH, Gratacap RL, Taggart JB, Hamilton A, Verner-Jeffreys DW, Paley RK, Rubin C, Bishop SC, Bron JE, Robledo D, Houston RD (2021) The nedd-8 activating enzyme gene underlies genetic resistance to infectious pancreatic necrosis virus in Atlantic salmon. Genomics 113:3842–3850 34547402 10.1016/j.ygeno.2021.09.012 PMC8682971

[CR40] Roelz R, Pilz IH, Mutschler M, Pahl HL (2010) Of mice and men: human RNA polymerase III promoter U6 is more efficient than its murine homologue for shRNA expression from a lentiviral vector in both human and murine progenitor cells. Exp Hematol 38:792–797 20685233 10.1016/j.exphem.2010.05.005

[CR41] Roy AL, Singer DS (2015) Core promoters in transcription: old problem, new insights. Trends Biochem Sci 40:165–171 25680757 10.1016/j.tibs.2015.01.007 PMC4340783

[CR42] Ruiz S, Tafalla C, Cuesta A, Estepa A, Coll JM (2008) In vitro search for alternative promoters to the human immediate early cytomegalovirus (IE-CMV) to express the G gene of viral haemorrhagic septicemia virus (VHSV) in fish epithelial cells. Vaccine 26:6620–6629 18840493 10.1016/j.vaccine.2008.09.048

[CR43] Samsing F, Alexandre P, Rigby M, Taylor RS, Chong R, Wynne JW (2020) Transcriptome response of Atlantic Salmon (*Salmo salar*) to a new piscine orthomyxovirus. Pathogens 9:807. 10.3390/pathogens9100807 33007914 10.3390/pathogens9100807 PMC7600774

[CR44] Sanjana NE, Shalem O, Zhang F (2014) Improved vectors and genome-wide libraries for CRISPR screening. Nat Methods 11:783–784 25075903 10.1038/nmeth.3047 PMC4486245

[CR45] Schaub M, Myslinski E, Schuster C, Krol A, Carbon P (1997) Staf, a promiscuous activator for enhanced transcription by RNA polymerases II and III. EMBO J 16:173–181 9009278 10.1093/emboj/16.1.173 PMC1169624

[CR46] Schaub M, Krol A, Carbon P (1999) Flexible zinc finger requirement for binding of the transcriptional activator Staf to U6 small nuclear RNA and tRNASec promoters. J Biol Chem 274:24241–24249 10446199 10.1074/jbc.274.34.24241

[CR47] Schramm L, Hernandez N (2002) Recruitment of RNA polymerase III to its target promoters. Genes Dev 16:2593–2620 12381659 10.1101/gad.1018902

[CR48] Shalem O, Sanjana NE, Zhang F (2015) High-throughput functional genomics using CRISPR–Cas9. Nat Rev Gen 16:5. 10.1038/nrg3899 10.1038/nrg3899 PMC4503232 25854182

[CR49] Sommerset I, Wiik-Nielsen J, Moldal T, Oliveira VHS, Svendsen JC, Haukaas A and Brun E (2024) Norwegian fish health report, Norwegian veterinary institute report, series 8b/2024. Published by the Norwegian Veterinary Institute

[CR50] Strømsnes TAH, Schmidke SE, Azad M, Singstad Ø, Grønsberg IM, Dalmo RA, Okoli AS (2022) CRISPR/Cas9-mediated gene editing in salmonids cells and efficient establishment of edited clonal cell lines. Int J Mol Sci 23:16218. 10.3390/ijms232416218 36555862 10.3390/ijms232416218 PMC9787932

[CR51] Sturm RA, Das G, Herr W (1988) The ubiquitous octamer-binding protein Oct-1 contains a POU domain with a homeo box subdomain. Genes Dev 2:1582–1599 2905684 10.1101/gad.2.12a.1582

[CR52] van Rijn CA, Jones PL, Evans BS, Afonso LOB (2021) Physiological and growth responses of juvenile Atlantic salmon (*Salmo salar*) transferred to seawater during different stages of smolt development. Aquaculture 538:736527. 10.1016/j.aquaculture.2021.736527

[CR53] Varshney A, Chahal G, Santos L, Stolper J, Hallab JC, Nim HT, Nikolov M, Yip A, Ramialison M (2021) Human cardiac transcription factor networks. In: Wolkenhauer O (ed) Systems Medicine. Academic Press, pp 429–453. 10.1016/B978-0-12-801238-3.11597-1

[CR54] Wang T, Wei JJ, Sabatini DM, Lander ES (2014) Genetic screens in human cells using the CRISPR-Cas9 system. Science 343:80–84 24336569 10.1126/science.1246981 PMC3972032

[CR55] Wang B, Wang H, Gao C, Liu Y, Jin C, Sun M, Zhang Q, Qi J (2018) Functional analysis of the promoter region of Japanese flounder (*Paralichthys olivaceus*) *β-actin* gene: a useful tool for gene research in marine fish. Int J Mol Sci 19:5. 10.3390/ijms19051401 10.3390/ijms19051401 PMC5983668 29738459

[CR56] Xiaohui Z (2008) Analysis on TATA-box, GC-box and CAAT-box in eukaryotic promoters. J Anhui Agric Sci 36:1380

[CR57] Zenke K, Kim KH (2008) Novel fugu U6 promoter driven shRNA expression vector for efficient vector based RNAi in fish cell lines. Biochem Biophys Res Commun 371:480–483 18452704 10.1016/j.bbrc.2008.04.116

[CR58] Zhu S, Li W, Liu J, Chen C-H, Liao Q, Xu P, Xu H, Xiao T, Cao Z, Peng J, Yuan P, Brown M, Liu XS, Wei W (2016) Genome-scale deletion screening of human long non-coding RNAs using a paired-guide RNA CRISPR–Cas9 library. Nat Biotech 34:12. 10.1038/nbt.3715 10.1038/nbt.3715 PMC5592164 27798563

