# Supplementary figures and images for: Exploring the Use of Alternative Promoters for Enhanced Transgene and sgRNA Expression in Atlantic Salmon Cells
Source: Mar Biotechnol (NY). 2024 Aug 30;26(6):1143–54. doi: 10.1007/s10126-024-10362-4 (PMC11541246; doi:10.1007/s10126-024-10362-4)

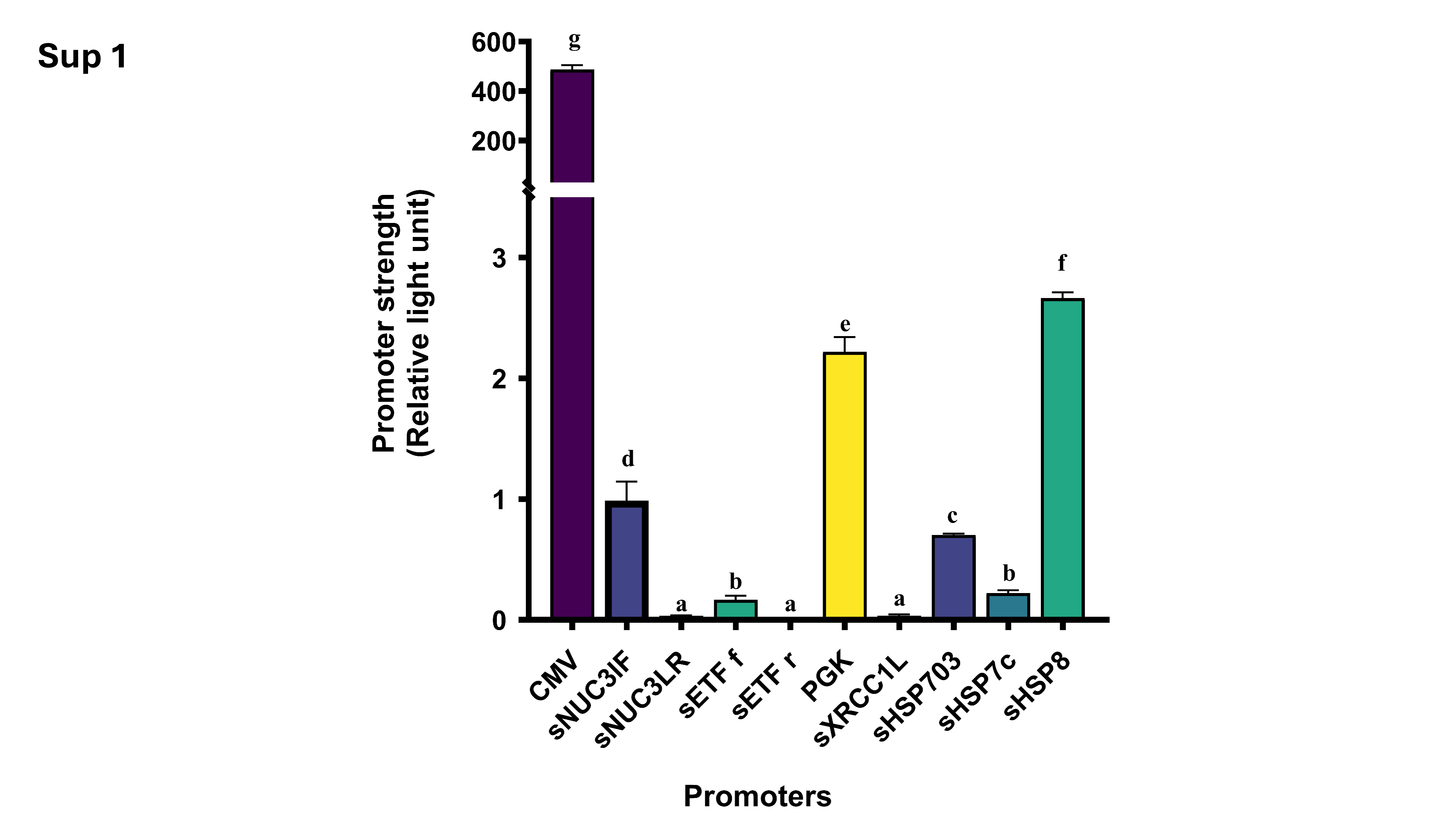

Supplement: Supplementary file 1 — Supplementary file1 (TIF 960 KB) [file 10126_2024_10362_MOESM1_ESM.tif]

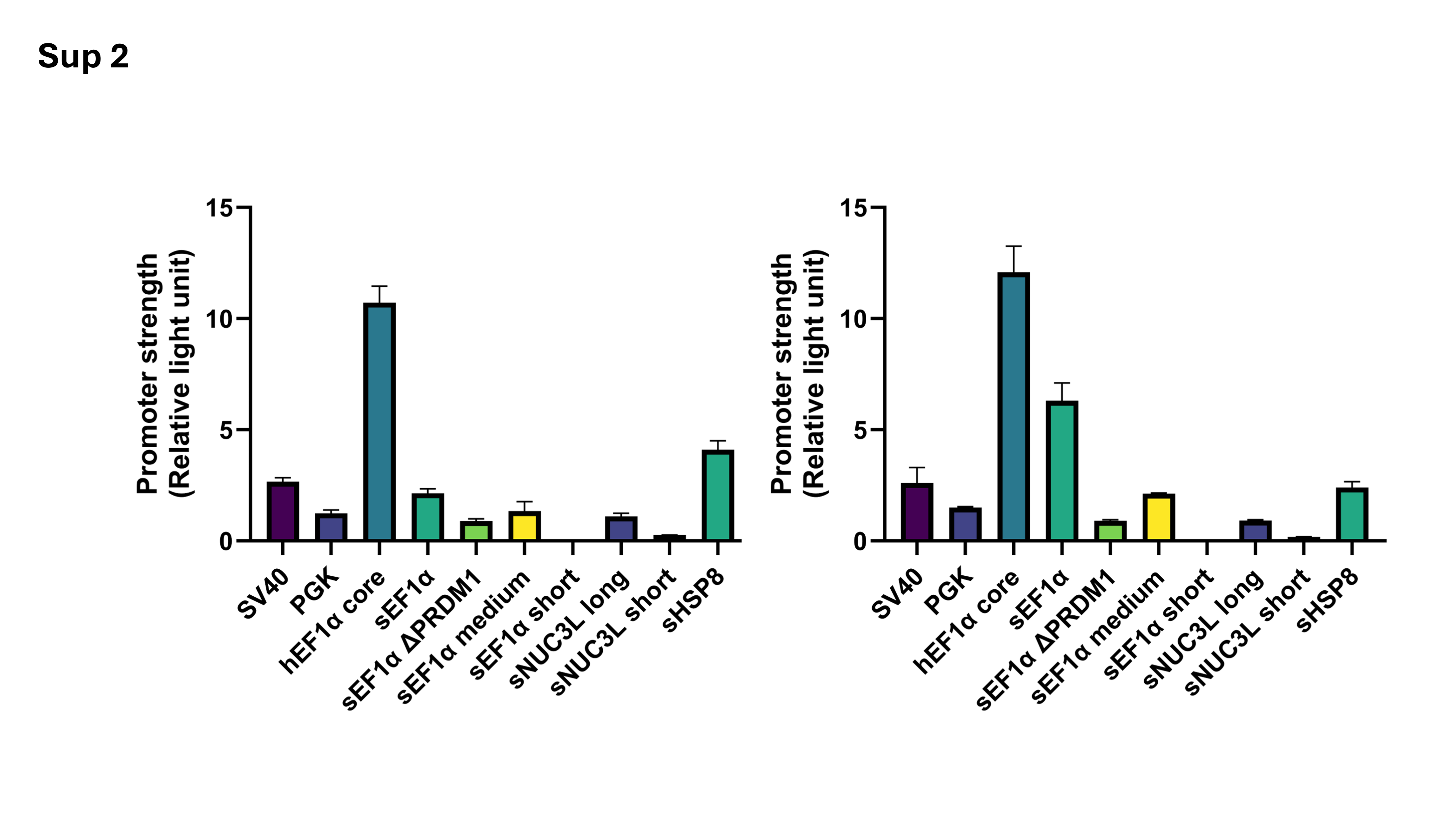

Supplement: Supplementary file 2 — Supplementary file2 (TIF 1403 KB) [file 10126_2024_10362_MOESM2_ESM.tif]

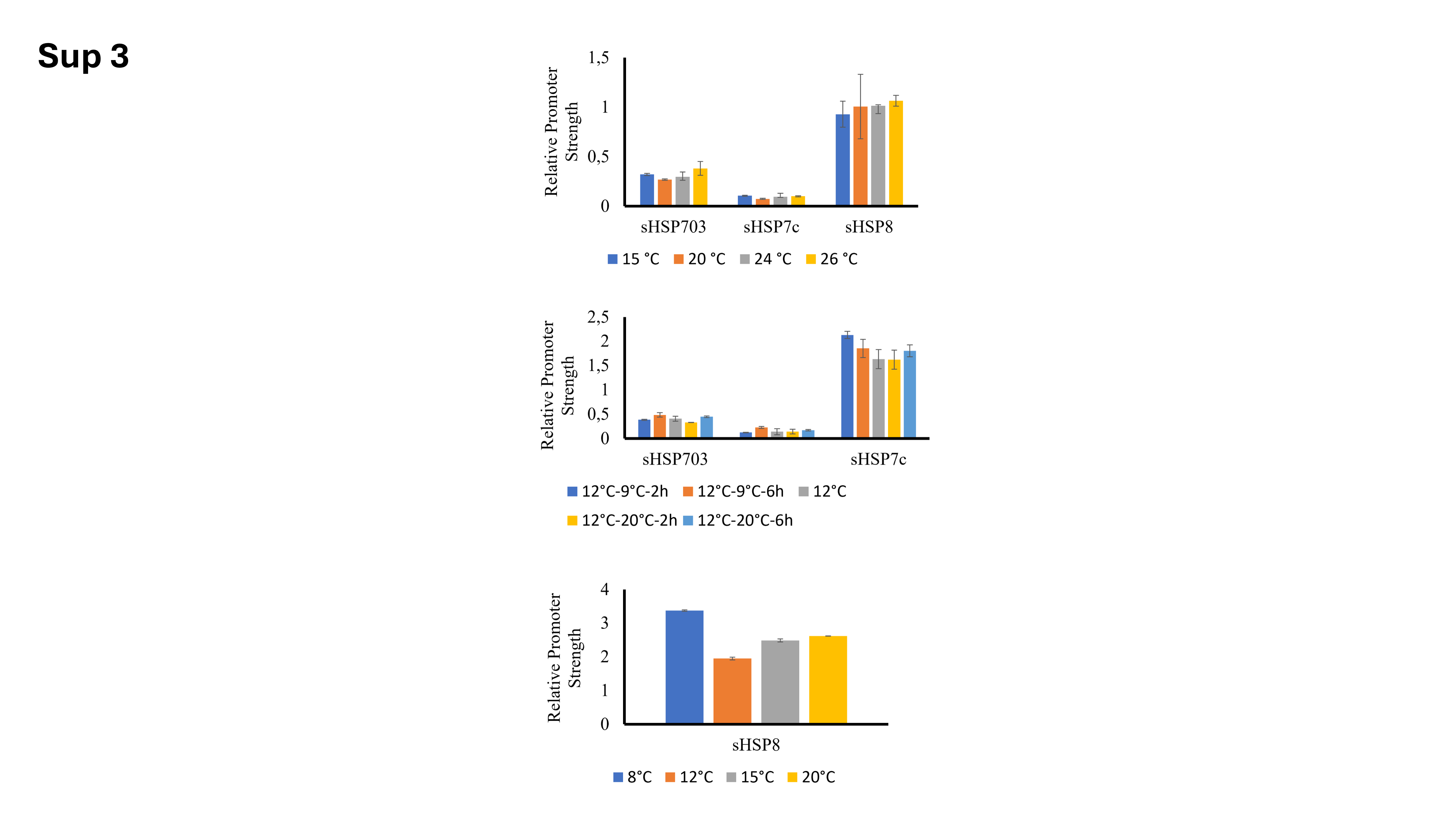

Supplement: Supplementary file 3 — Supplementary file3 (TIF 844 KB) [file 10126_2024_10362_MOESM3_ESM.tif]
