# Supplementary material for: Exploring the Use of Alternative Promoters for Enhanced Transgene and sgRNA Expression in Atlantic Salmon Cells
Source: Mar Biotechnol (NY). 2024 Aug 30;26(6):1143–54. doi: 10.1007/s10126-024-10362-4 (PMC11541246; doi:10.1007/s10126-024-10362-4)
Supplement: Supplementary file 4 — Supplementary file4 (DOCX 51 KB) [file 10126_2024_10362_MOESM4_ESM.docx]

| Serial | Promoter type | Promoter Name | Associated Gene Name | NCBI Gene ID |
| --- | --- | --- | --- | --- |
| 1 | Pol III - U6 | Human U6 (HU6) | RNU6-1 RNA, U6 small nuclear 1 | 26827 |
| 2 | Pol III - U6 | Mouse U6 (MU6) | Gm24019 predicted gene, 24019 | 115487555 |
| 3 | Pol III - U6 | Zebrafish U6 (ZU6) | BX511129.1 (Ensemble) | ENSDARG00000083520 (Ensemble) |
| 4 | Pol III - U6 | Salmon U6 (SU6) | U6 spliceosomal RNA | 123740086 |
| 5 | Pol III - U6 | Medaka U6 (EU6) | U6 spliceosomal RNA | 111948268 |
| 6 | Pol III - U6 | Tilapia U6 (TU6) | U6 spliceosomal RNA | 112847594 |
| 7 | Pol III - U6 | Fugu U6 (FU6) | U6 spliceosomal RNA | 115246710 |
| 8 | Pol II | NUC3l | 5'-nucleotidase domain-containing protein 3-like | 106573039 |
| 9 | Pol II | ETF | Eukaryotic translation initiation factor 4E transporter | 100196839 |
| 10 | Pol II | XRCC1l | DNA repair protein XRCC1 | 106605164 |
| 11 | Pol II | HSP70-3 | Heat shock cognate 70 | 100196655 |
| 12 | Pol II | HSP7C | Heat shock cognate 71 kDa protein | 100286711 |
| 13 | Pol II | HSP8 | Heat shock protein 8 | 100194521 |
| 14 | Pol II | EF1α | Elongation factor 1-alpha, oocyte form | 100136485 |

Table S1: Overview of promoters included in this study with associated gene names and accession ID of these genes

table S2: Sequences of oligos and primers that are mentioned in materials and methods

| Serial | Primer Name | Sequence | Purpose |
| --- | --- | --- | --- |
| 1 | lenti insert u6, gRNA check | CGGGTTTATTACAGGGACA | For sequencing |
| 2 | GFP_KO_qPCR_pLenti_Fwd 2 | GCGAGGAGCTGTTCACCG | qPCR (U6 Assay) |
| 3 | GFP_KO_qPCR_pLenti_Rev1,2 | ACTCGGTGCCACTTTTTCAAG | qPCR (U6 Assay) |
| 4 | Cas9_KO_qPCR_pLenti_Fwd 1 | ACCTATGCCCACCTGTTCG | qPCR (U6 Assay) |
| 5 | Cas9_KO_qPCR_pLenti_Rev1 | AGGATTGTCTTGCCGGACTG | qPCR (U6 Assay) |
| 6 | gfp_ko_fwd (sgRNA) | CACCGGGCGAGGAGCTGTTCACCG | gRNA |
| 7 | gfp_ko_rev (sgRNA) | AAACCGGTGAACAGCTCCTCGCCC | gRNA |
| 8 | slc45a2_ko_fwd (sgRNA) | **cacc**GGAACAGGCCGATAAGAC | gRNA |
| 9 | slc45a2_ko_rev (sgRNA) | aaacGTCTTATCGGCCTGTTCC | gRNA |
| 10 | slc45a2_KO_sanger_ss_g1_Fwd 1 | GGA ATC ATA CCC GGC CAA C | Edit check |
| 11 | slc45a2_KO_sanger_ss_g1_Rev 1 | CGC AAC GAC TAC ACA TTA TTA GC | Edit check |
| 12 | slc45_Ss_seq_R | CTCCTATGGTCATTGTAGTGGC | Edit check |
| 13 | EF1a core_fwd_kpni | TAGATggtaccGGGCAGAGCGCACATCGC | Luciferase assay |
| 14 | EF1a core_rev_XHOI | ATGATctcgagCTGTGTTCTGGCGGCAAACC | Luciferase assay |
| 15 | Ef1a_all_FWD_kpni | TAGATggtaccTCCAGAAAGCAGGTTTTAATAATC | Luciferase assay |
| 16 | Ef1a_Long_rev_XHOI | ATGATctcgagGGTTGCTTAATTCGTTTCTGAAAT | Luciferase assay |
| 17 | Ef1a_mid_rev_XHOI | ATGATctcgagGGTGTTACTGTGCGCTCAC | Luciferase assay |
| 18 | Ef1a_short_rev_XHOI | ATGATctcgagATCACACACAATTCAGCGAAAAA | Luciferase assay |
| 19 | Nuc3l_Long_kpni_FWD | TAGATggtaccGAGCCTGTGTTGGTATCTGAG | Luciferase assay |
| 20 | Nuc3l_Long_rev_XHOI | ATGATctcgagGACTAGTCGCCTTCGGTTTGT | Luciferase assay |
| 21 | Nuc3l_short_rev_XHOI | ATGATctcgagGGTGAAGTAGTAATGTTTCTTCTTCA | Luciferase assay |
| 22 | SV40_FWD_kpni | TAGATggtaccGGTGTGGAAAGTCCCCAGG | Luciferase assay |
| 23 | SV40_rev_XHOI | ATGATctcgagTTTGCAAAAGCCTAGGCCTCC | Luciferase assay |
| 24 | Hsp8_FWD_kpni | TGATGGTACCTCAGATCCTTATCTAGCGCACC | Luciferase assay |
| 25 | Hsp8_rev_XHOI | ACTGCTCGAGATGCTGATGTTGATCGGAGTTG | Luciferase assay |
| 26 | Pgk_FWD_kpni | TGATGGTACCGGTAGGGGAGGCGCTTTTC | Luciferase assay |
| 27 | Pgk_rev_XHOI | ACTGCTCGAGCGAAAGGCCCGGAGATGAG | Luciferase assay |
| 28 | insert check_pgl4.10 | GAATCGATAGTACTAACATACGC | For sequencing |

Table S3: Potential transcription factor binding sites of pol III and pol II promoters and their locations are indicated in the table above. The MEME suit (Motif-based sequence analysis tools) was used to identify the motifs. For pol III, consensus sequences for CCAATYA, Octamer (OCT), Proximal Sequence Element (PSE), SPH, and TATA box were obtained from already published paper and scanned against U6 promoter in FIMO (Find Individual Motif Occurences). The potential motif matches were filtered by setting the p-value <0.001. For pol pol II, JASPAR2022 CORE vertebrates' non-redundant database was used against targeted promoters. The potential motif matches for pol II promoters were filtered out by setting the p-value <1E-6.

| **Motif_id** | **Species**  **name** | **start** | **stop** | **strand** | **score** | **p-value** | **q-value** | **matched_sequence** |
| --- | --- | --- | --- | --- | --- | --- | --- | --- |
| PSE | FUGU | 96 | 113 | + | 8.46341 | 1.98e-05 | 0.0428 | CTGACGCTGTATTAAATG |
| SPH | FUGU | 226 | 244 | + | 4.86585 | 0.000225 | 0.12 | TTTCCCATCAGCCCCCTGT |
| TATA box | FUGU | 340 | 346 | + | 7.21512 | 0.000988 | 0.264 | TCTATAT |
| TATA box | FUGU | 342 | 348 | + | 7.21512 | 0.000988 | 0.264 | TATATGT |
| SPH | FUGU | 401 | 419 | + | 3.43293 | 0.000541 | 0.165 | ATTCCAACCATGACCCAGA |
| CCAATYA | Human | 4 | 12 | + | 6.60976 | 0.000516 | 0.71 | GGCCTATTT |
| OCT | Human | 29 | 36 | + | 14.7744 | 2.06e-05 | 0.0225 | ATTTGCAT |
| PSE | Human | 184 | 201 | + | 03.61 | 0.000139 | 0.0795 | CTTACCGTAACTTGAAAG |
| TATA box | Human | 219 | 225 | + | 7.21512 | 0.000988 | 0.264 | TTTATAT |
| TATA box | Human | 221 | 227 | + | 12.407 | 0.000114 | 0.0815 | TATATAT |
| TATA box | Human | 223 | 229 | + | 7.21512 | 0.000988 | 0.264 | TATATCT |
| SPH | Medaka | 107 | 125 | + | 9.41463 | 3.97e-05 | 0.0282 | TTTCCCAGAATCCTTCCAA |
| OCT | mouse | 85 | 92 | + | 14.7744 | 2.06e-05 | 0.0225 | ATTTGCAT |
| PSE | mouse | 247 | 264 | + | 7.09146 | 6.53e-05 | 0.0706 | CTCACCCTAACTGTAAAG |
| OCT | Sal | 6 | 13 | + | O9.22 | 0.000352 | 0.256 | ACTTGCAT |
| SPH | Sal | 15 | 33 | + | 19.378 | 2.11e-07 | 0.000452 | TCACCCAGCATACATTGCA |
| SPH | Sal | 180 | 198 | + | 1.28659 | 0.000823 | 0.22 | CCAACCAGGATCCGAAACG |
| TATA box | Sal | 214 | 220 | + | 7.21512 | 0.000988 | 0.264 | TCTATAT |
| TATA box | Sal | 216 | 222 | + | 12.407 | 0.000114 | 0.0815 | TATATAT |
| CCAATYA | Tilapia | 96 | 104 | + | 6.57927 | 0.000636 | 0.71 | GTCCAATCA |
| SPH | Tilapia | 136 | 154 | + | 9.67073 | 3.18e-05 | 0.0282 | TTACCCACAATGCCTCCGT |
| SPH | Tilapia | 251 | 269 | + | 3.87805 | 0.000436 | 0.155 | CGACCCAGCAGCACCCAGA |
| PSE | Tilapia | 326 | 343 | + | 3.46341 | 0.000147 | 0.0795 | GTAACCGTGACTCACACA |
| SPH | Tilapia | 337 | 355 | + | 4.33537 | 0.000334 | 0.143 | TCACACAGCTTCCAGGACA |
| TATA box | zebrafish | 271 | 277 | + | 12.407 | 0.000114 | 0.0815 | TATATAT |

| motif_id | motif_alt_id | sequence_name | start | stop | strand | score | p-value | q-value | matched_sequence |
| --- | --- | --- | --- | --- | --- | --- | --- | --- | --- |
| MA1859.1 | FoxA-a | ETF | 286 | 305 | + | 19.8468 | 5.66e-08 | 0.000369 | CTGCTATGTAAACAAACCAT |
| MA1865.1 | FoxG | ETF | 1328 | 1347 | + | 14.1065 | 1.23e-07 | 0.000763 | GCGTGTTTGTTTGCTTGGTT |
| MA1343.1 | BZIP52 | ETF | 1673 | 1686 | + | 16.7231 | 2.99e-07 | 0.00203 | TTGACAGCTTGTTA |
| MA1823.1 | Zm00001d027846 | ETF | 601 | 612 | + | 15.56 | 3.69e-07 | 0.00248 | AGAAAGAAAAAA |
| MA0846.1 | FOXC2 | ETF | 290 | 301 | + | 15.6471 | 4.18e-07 | 0.00276 | TATGTAAACAAA |
| MA1527.1 | NFIC | ETF | 1112 | 1128 | + | 18.8448 | 4.46e-07 | 0.00302 | GTTGGCTGTTTGCCAAA |
| MA0832.1 | Tcf21 | ETF | 133 | 146 | + | 18.7867 | 4.62e-07 | 0.00313 | AAAACAGCTGTTGA |
| MA1898.1 | Meox | ETF | 889 | 908 | + | 12.1102 | 5.03e-07 | 0.00299 | TTTAAATTAATGATTTATAA |
| MA1606.1 | Foxf1 | ETF | 291 | 301 | + | 13.7903 | 5.12e-07 | 0.0034 | ATGTAAACAAA |
| MA1528.1 | NFIX | ETF | 1112 | 1128 | + | 17.6327 | 7.87e-07 | 0.00533 | GTTGGCTGTTTGCCAAA |
| MA1206.1 | ARF2 | ETF | 1417 | 1426 | + | 2452939 | 8.27e-07 | 0.00565 | AGCCGACAAG |
| MA0510.2 | RFX5 | ETF | 500 | 515 | + | 16.6034 | 8.59e-07 | 0.00583 | CGTTACTGTAGCAACC |
| MA0699.1 | LBX2 | ETF | 1619 | 1628 | + | 12.0678 | 9.17e-07 | 0.00611 | GCCAATTAGC |
| MA0047.3 | FOXA2 | ETF | 291 | 301 | + | 14.021 | 9.98e-07 | 0.00653 | ATGTAAACAAA |
| MA1862.1 | FoxD-b | HSP7C | 31 | 50 | + | 16.25 | 4.22e-08 | 0.000237 | CTATTTTTGTTTATTTTTGA |
| MA1869.1 | FoxK | HSP7C | 31 | 50 | + | 14.4653 | 1.23e-07 | 0.000708 | CTATTTTTGTTTATTTTTGA |
| MA1385.1 | AT2G40260 | HSP7C | 291 | 305 | + | 16.8308 | 1.39e-07 | 0.000805 | TTAAAAAATTCTCTT |
| MA1863.1 | FoxE | HSP7C | 30 | 49 | + | 14.2976 | 3.25e-07 | 0.00189 | GCTATTTTTGTTTATTTTTG |
| MA1873.1 | FoxQ | HSP7C | 32 | 51 | + | 1143180 | 9.77e-07 | 0.00558 | TATTTTTGTTTATTTTTGAC |
| MA0508.3 | PRDM1 | HSP8 | 1265 | 1275 | + | 15.6829 | 2.37e-07 | 0.00151 | TTCTTTCTCTC |
| MA0463.2 | BCL6 | HSP8 | 92 | 107 | + | 17.1579 | 8.69e-07 | 0.00556 | TCGCTCTCCAGGAATA |
| MA0139.1 | CTCF | NUC3L | 109 | 127 | + | 17.2131 | 8.06e-07 | 0.00545 | CATCCAATAGGTGGCGGCA |
| MA1734.1 | AT1G19040 | ssEF1α | 1219 | 1234 | + | 19.7869 | 7.56e-08 | 0.0005 | CTTGTTCCCCAAGCAA |
| MA1274.1 | DOF3.6 | ssEF1α | 1085 | 1105 | + | 18.6515 | 1.84e-07 | 0.00099 | TTGACTTTTTTTTTTTCTCTC |
| MA1630.2 | ZNF281 | ssEF1α | 777 | 788 | + | 20.0337 | 2.22e-07 | 0.00151 | GGGGGAGGGGGA |
| MA1317.1 | WRKY50 | ssEF1α | 1083 | 1095 | + | 18.0923 | 3.64e-07 | 0.00236 | AGTTGACTTTTTT |
| MA1789.1 | NAC071 | ssEF1α | 1219 | 1233 | + | 17.1148 | 4.49e-07 | 0.00297 | CTTGTTCCCCAAGCA |
| MA1380.1 | TCX6 | ssEF1α | 1138 | 1152 | + | 18.0896 | 4.6e-07 | 0.00279 | TTTTGAATTTTTTTT |
| MA1313.1 | WRKY7 | ssEF1α | 1081 | 1094 | + | 18.4769 | 5.15e-07 | 0.00321 | TCAGTTGACTTTTT |
| MA1318.1 | WRKY27 | ssEF1α | 1081 | 1093 | + | 18.0615 | 6.4e-07 | 0.00409 | TCAGTTGACTTTT |
| MA0611.2 | Dux | ssEF1α | 249 | 264 | + | 18 | 6.98e-07 | 0.00436 | TTGATTGAATCAGAAA |
| MA0508.3 | PRDM1 | ssEF1α | 442 | 452 | + | 15.6098 | 7.19e-07 | 0.00229 | TTCTTTCTCTT |
| MA1300.1 | WRKY6 | ssEF1α | 1083 | 1101 | + | 17.0286 | 7.29e-07 | 0.00473 | AGTTGACTTTTTTTTTTTC |
| MA1012.1 | AGL27 | ssEF1α | 1130 | 1143 | + | 17.36 | 9.67e-07 | 0.00567 | CTTTCTTTTTTTGA |
| MA1274.1 | DOF3.6 | ssEF1α | 1130 | 1150 | + | 16.7727 | 9.82e-07 | 0.00264 | CTTTCTTTTTTTGAATTTTTT |
| MA1933.1 | ELK1::SREBF2 | XRCC1l | 156 | 171 | + | 18.5484 | 3.1e-07 | 0.0021 | ACCGGAAGTTACGTCA |
| MA1955.1 | FOXO1::ELK3 | XRCC1l | 152 | 165 | + | 17.5658 | 5.8e-07 | 0.00394 | ATCAACCGGAAGTT |
| MA1954.1 | FOXO1::ELK1 | XRCC1l | 152 | 165 | + | 17.2551 | 8.7e-07 | 0.00592 | ATCAACCGGAAGTT |
| MA1952.1 | FOXJ2::ELF1 | XRCC1l | 152 | 165 | + | 16.0484 | 9.95e-07 | 0.00676 | ATCAACCGGAAGTT |

| # FIMO (Find Individual Motif Occurrences): Version 5.4.1 compiled on Aug 25 2021 at 17:37:39 |
| --- |
| # The format of this file is described at https://meme-suite.org/meme/doc/fimo-output-format.html |
| # fimo --oc . --verbosity 1 --thresh 0.001 --norc motifs.meme sequences.fa |
