# Supplementary material for: Exploring the Use of Alternative Promoters for Enhanced Transgene and sgRNA Expression in Atlantic Salmon Cells
Source: Mar Biotechnol (NY). 2024 Aug 30;26(6):1143–54. doi: 10.1007/s10126-024-10362-4 (PMC11541246; doi:10.1007/s10126-024-10362-4)
Supplement: Supplementary file 5 — Supplementary file5 (DOCX 20 KB) [file 10126_2024_10362_MOESM5_ESM.docx]

Supplementary file S1. Sequences used in the analysis of promoter activity.

>Human U6

GAGGGCCTATTTCCCATGATTCCTTCATATTTGCATATACGATACAAGGCTGTTAGAGAGATAATTAGAATTAATTTGACTGTAAACACAAAGATATTAGTACAAAATACGTGACGTAGAAAGTAATAATTTCTTGGGTAGTTTGCAGTTTTAAAATTATGTTTTAAAATGGACTATCATATGCTTACCGTAACTTGAAAGTATTTCGATTTCTTGGCTTTATATATCTTGTGGAAAGGAC

>Mouse U6

GATCCGACGCGCCATCTCTAGGCCCGCGCCGGCCCCCTCGCACAGACTTGTGGGAGAAGCTCGGCTACTCCCCTGCCCCGGTTAATTTGCATATAATATTTCCTAGTAACTATAGAGGCTTAATGTGCGATAAAAGACAGATAATCTGTTCTTTTTAATACTAGCTACATTTTACATGATAGGCTTGGATTTCTATAAGAGATACAAATACTAAATTATTATTTTAAAAAACAGCACAAAAGGAAACTCACCCTAACTGTAAAGTAATT

>Zebrafish U6

CACCTCAACAAAAGCTCCTCGATGTCACACAGGAAGTTCAGGAACTTATCCAATCACTCTAAAGAAACGGCCTGTTTCCTTCGCATACGCTTACAGCTCCAAAACTCTACGGTAAACCTACATAAACTGCTGGTTTTCAAATTTTAAAGAATTTAAGGGTTTACAGGTTTACTACTACACAGTGATTTACTGACACATGTAGGTGTAAATGAGTTGAATAAGTAAGTAAGCTATATACCACACATGAAACACATACCCAGAAGTCACTGGTATATATAGCCGTCCTCCAGACTCCCA

>Salmon U6

AGTGTACTTGCATATCACCCAGCATACATTGCAACTTTCTGAGGCAGAGGCTAGAACATTACAACACACAAGGTAGCCATTAAACGATACATTATTATCCCTACCAGGTTCATGTCTATACAATATCCTGTGGGAATCTTCTATGGGTGGTTTTTGAGCCTCTGAAGGTCTGTTTGACCCCAACCAGGATCCGAAACGTCAGGGGGGTTGTGCTCTATATATGAGGGCCTCTCCTATGTCTGTATT

>Tilapia U6

CTGAAGTATACTATGTGCCGAATTTCCCACCAAGTGGTAATGAGCAATGTTTGTTATTATGGCTCACTATTACTTTTGTTCTGCTGCCTTCAGCCGTCCAATCAGAAGCGTGTGAGTAAAACCGGTCTGTGTAAATTACCCACAATGCCTCCGTGTGTCTCACTCACAGGCTCAAAGTGAAAGCAGAGAATGGGGAACAGACTCAGACTGGCGCTCTCTGAGCCTCTGTGTCTTCTTACAGATGTATAACCGACCCAGCAGCACCCAGACATGTTTCTGCTGCTGTTTCCCATCAGTTTAAACGCAGTTTAAGCGTCTTCCCCCTGTAACCGTGACTCACACAGCTTCCAGGACAGAGGGCTTTAAATTGCTCCCGGGTCCTTGGAGCTGTCG

>Fugu U6

CACTGGATCCATCTGACACTAAGAACATTCCAATTTTTGTCACTGGATCTATCCGATGCTTAGTCATTACATTCCTATTTTGTCAGTGAATCTATCTGACGCTGTATTAAATGTTTGTGGGTATTTACCCCAAATTTACACGGTTGTTCAAAGGATTCCACCCACTGGAGAGATTGTGAAACTGCAACCACATGCTGTTAAAATGGAAACAGGAAGAGGTTCACATTTCCCATCAGCCCCCTGTGAATCAAAACAAACTGCTAAGAATTGGACCGGGACGGGTGACATTATGTCCCAGTGAGTCAGTACTGATACAGAATATTCAATTTAAGACTCCACTCTATATGTTGGCCTGAATGGAAACTTGAGAGCCACTGTGTTCTTGTCATGGCGGCTCAGTATTCCAACCATGACCCAGAATGGTGTTGGAGCATCGGCCTACATATACCACGTAGATGTTGCTCTCTGTT

>Medaka U6

GTGGGGATTGGTAAAGGTCCTCCTGGAGGGGTGTTGACTTGACCTTTTGTTCTGGCGCTTCCGGGAAACCAGTCGCACTCGTCTGACGTGACAAAACCCCCCTTCTTTTCCCAGAATCCTTCCAAGAAGTTTCTCACAAAGTCAGAAAAAATGTAATTTTAATCTGATCAGAAACATTTAAGTCAAACTTTAAATGGAGATGGTGAACCTGAGACGATCCTGTGTCATGGGAGAAACAGATGATCCTTTTTGGTGTTTTGTCATCATTGTGGTTCCCTTTAAAGTCCACTGCGTTCCCTCAGATCACACCTCATGCTTGAGAGGAGTAGCAGAGGGGAGGGTTTAAAAAGCACTCAGAGACTTTGGCTCATC

>sNUC3L

GAGCCTGTGTTGGTATCTGAGTAGACATTTGAATCCGCTTCAAGGAGGAAAGCTTTAATTTTTGTTCCCCCTTCCCCTTCCCCTATTAAAGTTGATCCGTTGTCACCCCATCCAATAGGTGGCGGCACGCACCTCTAACGTTTGTTTGCAAAACACTATACTACTTTTTAAGAAGAATAAGGTCTGTCATTTTGAGTGTTGACCATAGACAATGATAGAAGCCTCTAAGTGGCCAAAACGCTATTTATTAGCATGGGCAGCGCCATTGAGGGCTTCCACCATTATAACGTAGTCGACTGGGTGGTACTTCCAACTTCATTGGCTGATCCCTCCTGGTGACCCTATTGGAATAATGTCCAACTGGGTCATCAGCCAATCGTGAAGAAGAAACATTACTACTTCACCATGGAGACAGCCTCAATGGCTCTGCCCATGCCATCGCAGACGCTATAATTTCACAGATACAACAATTAGTCCTCTATCTATATCTACGGTGTTGTGAGTGGAACCAAGGAAGTGACGTAGCCACGGAAGAGCATGGACCGATTCGGTTTCTGTATTTACAGAAGAGTGCTCTAACCGAGTGCATTTACCTAGAGCCCACGACCTACACGCTGGTTACTGACAAACCGAAGGCGACTAGTC

>sETF

CATTTTTAGGGCTGAGTAAACCCTCTCGTTTAGCCCTTTCCCCTGTGATTTGTCGCCACATATGGTTTGTTTACATTTCCTCGGTTGTGGTTAGGGGTAGGCCTACATCTCCATCCAGTGGTCGCGTCGGGGAAAACAGCTGTTGACAACAGAAGCATTAGCTAACCCTAACTCTTTTCATAACCTTAACCTCATAACCTGCTACATTAATTCACATATCCTGCTGCGTTAGTTCTCGTAACCTGCCACGTTAATTATCCTAGCCTGCGACGTTAATTATCCTGACTGCTATGTAAACAAACCATCTGTGCCCAAAGTCATCAGTATCACCACACCTGTTAAATTAGCTATCTTTGTGCGATTAATATAATTTATTTTTAACAAATTGTTTGATGCCTCTGTCATACCCAGTGAAATTAATAAACATGCGGATCTTTATGGTGGAACCAATGACGGTAAAGTTACCACCACTCACAGTGACCGCGTGTTGTTTTGCAAACGTTACTGTAGCAACCCTCTGTACAAATACTGAACTGATGAGCGTGATGTAAAGCACATCGAACTTTACCCTAAAATGACAGGGTGTGGTATCATTTACAAAGAAAGAAAAAAGGTGGATGTGTTGAGTGCCATTTGGTTGGCCTATATGTCAAATTATTAATTAAGGAAAGGGCTAGTTTGTTTAAAAAAGTACATTGAAAGTATGACAATTATCGATCAGTTTGGGCTTTCATCGATCCGCTTTGTAATATCAACAATTAGCTGGCCATACATAATGCCTGATATAAAAATACTCACTCGTAAAATACAAGCAGTCGTTGAAATTTCATCTAAATTCATATATATTAATTTCGTAGTCAAAAGAGACTATGTCAAAGACAATAATGGTTTAAATTAATGATTTATAATATTTTCATATGTTTGAGTAAGGCACTAGTGATGGGCACTATATAATCCTACGAAGGACCCTCATTCAGAAGCGGTGATCGGAGAAAAATCACTGCTACGACTGAGAAAAGAGACCAAAGAGAAGTATATTTCGACATTCGAATACAATCCCAGCAAGACGCTAGTATACGTTTGGATTTCTGTTCGTAGAAATATCACGGGCGTTGGCTGTTTGCCAAAATAACAACCCTAGCTGTTTAGAAAAACGTATCGTACTTATTGGAAGGCTTTATCTCCAGCAAGCTAGCTGGCTAACTAGCCATAGAGCTAGCTTAGCTAGCTTCTGAGTTGGCTTCAAACGTTTTCTAAACAGCATTGTAAGTTAATTGTTACCTTCTACATGTATAAGTATCTGCAATTTATCCAGAGTGATATGATGGCGTGTTTGTTTGCTTGGTTAACGTTACATATGCACTCGGCGTTTATCAATTGAATAGCCGAACACAAACGTTAACTAGCTATCAGATTAGCCGACAAGTCGGCTTTCAGGCTAACTAGCTAAACTAAAGTTTTGGCAGCTAACTAGCTAATGTTCGCTAACTAGTTACCAGTAAACCAAACACAACGGTCGTGTAATTTAGCTAATTGTCCGCCGTCCATCGAAGATTTGACTAGGGTCTTGTAAATTGGCATCAACATATGCATTAGCTACATCTGCCAACGCAGTTTTGCCAATTAGCTAGTTAACTACGCAAATATGCTAACTAACGTTAGCTAACTTAGATTGACAGCTTGTTAATTAATTCTGGCCAGTTTCCTAGCATGCTAACGATAGCCAATTT

>sXRCC1l

TTCTCCCCATTTCCCTTTTTTGTGAACAATGTGCAATGCTCTTTTCGAACTGTTAAAGGCAGAAATACGCAACAAAGAGTCTAGTCGGCCGTAAAACCACACGAAGAAGAATAAGAAGAAGCGTCAACTGTTTTGATTGGCTAAATAAAATATCAACCGGAAGTTACGTCAGAGATTATGAATTGAGAGGGATTTTGGCGTGAAATTCAGGGGGCACGGTTCAACCTCCAAATACGTTTCGGCTGTTTTTGTCCGCCTTTACATTAGAATAAAATTCACAGGGAAGTCAAATACAATGCATAAAACACAGAAATGTCAAACACGGTACTTCTATTAAGCATTTAGAGGTGTTTTGCTTTCTATAAACGTGTTCCGTTGTTTGCTACCCAGGCAATTGCTAACTAGCTAGCAAGCGTAGAGCTTTTGATTAACTGAAAAAACACAAATAAAGCGCATCACTTTTTTGACAAGTCAAGTGACTGGCGCAAA

>sHSP70-3

TAAGCCCTTGGTGAATGCTTTCATGGTTAGTGTAGTATTTTCATGGAGCATGCTATGAAAGGTTCAGACAACTATATATAAAAATATGCTTAATTTATTCCAAAATTCTTTCAATTCATTTCATCATGTAATTTACTGTACAAACTAGAATGTGCTTTGCTGCTTGCTGTCCTGCTTTTCTAATGAATGTGAATCCACAGCCCGGCTGCTGGTAGGAGATATCTGTAGTCTTCTCACAGTGCTTATCTCACTGAACAGCCCCCAGACCATGTGAATCAAATTAAACATGCCCTGAGAAAACCTCCTTACTCTGTGAACAGGACAAACTTGTATCACATTTTTTTTATAGAACGGGAATTAAGATAAAGTGTAGAGAGAAAATACAATAGTCTTTTCTAATTTCTATTTTAATTCACTGAAAACGGGCTAACAGCAATTCAGCTAACTCCTATACCAGATCAAGTTAGAGTTGGCAAGCACTGAATGGGCACATGCAGGCAGCTACATGGGCCTAGAACTTTCTCTGGCTGAGTCACCTGATTCCCTGATGGAATCTCAGACAGCACACAGCTCACTGTCTGTGGCTATATACTCTGGAGAGACCTGACACTCGTCCTCTTAGTCTTTCTGTCCAGGAAAGGGAGAGGAGTGCTTCAGTGGAATTGAGCAGGCGGTGTTCTGTGGTTTGTGTGGTAATCTTTGAAGCGTTGC

>sHSP7C

TTGCTTGAGAAATTGCTCTTTGCTAAAAAGCTATTTTTGTTTATTTTTGACCTTTTAAATGGAAACCAGTCACAGTAAGGTACTTAATTGTTACCCAGAAATTATTTGATTTTTAGATGAAAAAAGCTGAATTGGGCCTTTAACAATTTACTTTTTTAAGTAAAGCTTGCATTCAACTAACTGCGCCTCCATGTGACACACAAGCTTCCATTCCCCCTGTCACAAGGGGATTTATGGCTGATTTACAGTCAACCTTGTTGCTTTATTAGGCACTTGCTATAGTATAATTTTTAAAAAATTCTCTTGAGAAGGGAAAAATTGTCTTTCATTAAGTTGAACATGTGAACTTTATGACAGAATGTTATAATTAGGTGAAATAAAACTGGGGGTTTTCTCAACAAGTTACACTACTCAATTTATGGAACAGTCCAACACTGTTTCTCTAAAAATAGGAAACACTATAGCAACTAACTTATCTACTGTGGACATTTTTTAATTGGTTCATTTGAGCTCGCGATGAAGCAACTGTTGGCAGTTGATTGGCTAGCTACATCGCTCAGTTTGTTCTAGACTGTTCTGCGGAAATCACCAGCCCGGTCAGTTTCTGCGAGGATAGAGTCCTGCTGTACCATTCAGGGTGAAGCGACATTTTCAGGCTGATTTG >sHSP8

TCAGATCCTTATCTAGCGCACCTAACTAACTGGTTGATAAGTTGAATCACGTTAGTTACTGCTGGGTTTGGAGCAAAAACCTACAGGATGGTCGCTCTCCAGGAATACAGTTGGTCATCCCTGCAATAGTGATTTGGGATTTGTAAAAAATAAATAAAAGTAGAGGCCAATAAATCATAAAATAATCTGAACCAATTATGCTAATCTGTTATGTTAAATATCCGTTATAATACACCCAGAAATGTTTAAGCCAAAAGACCACCTGGAACGCACCCATGTAGACCTGTGACGCGGCTGCGGGCTAACAACGTAACCACAGCCTAGCAATGAACGCACCCTTGTCGACCTGCACCCTGGAACGCGCCCGAGGGCTAGCAATGTCAGTAAAGCTAGTCAGCTGGGCCACGAGTTGCCGAAGCTCGTTCAGAGTTTAAAACTATGTTTTAAATAATTTAGGAACTGCAAATGCAATGCTCACTTATAATTTAATAATTAAAATTTTGCTTCCTATGGCGGGCAAGGGAGGTCTGGTTGTCGGCCATGCTGGAAATGTGATCTCGCGTTATTGTGTCATTGTCAATATAGTACATCTACACCACGACATGAAACGTTGATTTGAACCTTCTGACCCACTTGTGAATCAGGCGGGCCCCATTGTCTGATTTAAATCGCATGATTTCTGATGATAGTTGTCGAGTACATCGACTGATTGCAGTATATCGACTGATTGTTGTCGAGTATATCGACCTCATCCCCATACTGTATTTATGAATTTATCTTGCTCCTTTGCACCCCAGTATCTCTACTTGCACATTCATCTTCTGCACATCTACCATTTCAGTGTTTAATTGCTATATTGTAATTACTTCGCCACCATGGCCTATTTATTGCCTTAACTCCCTTATCTTACCTCATTTGCACTCACTGTATGTAGACTTTCTGTTTTATTTTTTTTCTACTGTATTATTGACTGTATGTTTTGTTTATTGCCATGTGTAACTGTGTTGTTGTAAGTGTCGAACTGCTATGCTTTATCTTAGCCAGGTTGCAGTTGCAAATGAGAACTTGTTCTCAACTAGCCTACCTGGTTAAATAAATGTGAAAAAAAAAAATGTATCCGGTGCGAACGCAACCCGACGTCTTCATAGGCAGTGGGAGGGAGCAACAGCCTGTGTTTTTATTGGTATTTTGGAATGTCAAGATGAATCCTGAGCTATGATTGGTTGGCTTGCCGTTTATATAGTAATGGTAGACTTATCCTTCCTTCTTTCTCTCATAACACACAAGGCGAAGAAAGGGAGGCTTCGCCATTGTTCAACTCCGATCAACATCAGCAT

>sEF1α

TCCAGAAAGCAGGTTTTAATAATCTCACTCAGGGTTATGAGACAACGGGGATTGGTTTGTTGAGATATCTAACCACACTCAACATACAGTGGGGTAATCTACTGTCAGATTTTTGTTAAACTCTTGTCAATTGAGTTATTGAATGAGTGTCTGGTCATTTTTTTCTCGTGCCAATAAAGAGTGTACTTGACCACATGTGGTTATTTAATCAATTTATCAATTACCTAGCTATATTGTTTTTAAACATTTTGATTGAATCAGAAAGGATTTCGTTCAAAATTGGAAAATGTTTCAAACATCATGACGTTTCACGACGGACGGGCATAGTTTTTCGCTGAATTGTGTGTGATTTGAGGGTTCGAGTAGGGAGGGGGAAGGGAAGCGGGGACGCAACCCAGTCTAGCCACCTCCCAGTGTATAAAAACTCAAACTCAACCAGCATTCTTTCTCTTTTTGTTACCTGGGTAGGGGCATCAGCAGTGGTCGAGTGAGCGCACAGTAACACCGGTAAGCGCGTTTATCCTATTTAGCCTCTAGCGGTTTCAAAAACAGGCCTAACATGTTAGCACGTTTGTTTTTGTAACTTGTTAGACACATTGCGAAAACGTAAGCTATTTCCGATAGTATTTAATGGTGTGGACAATGTTTCAATTAAAGTGAATTGGATGGTGTTGGGATGCTTTAACGTTCATTTTTAAGTTGGCTTATAAAATGGCGATGTGGTTTTAGTGAACTTCCAAGTTATATTTTGCCGGGAGAAAAAACGTTTTTCAACTGGGGGAGGGGGAACTGGGGGGGGGGGACAAAGGATACTAAAGCGGTGGCCTGTGAGGATGAGTAGGTAGTTAAATGGAATGTCTTCCATGATCTGAACAACTGCACGAAAACCCAGGCGACTTTTGTCTAGTAAGGATAAATTCCTTCTGGATATTTGGAGTTCTTTCTGTAACTCTATACGATGTGGCCTGGAAGTAACACGTGTCGCTTTTGGCCCATGGCCGACTGAGGCCTAACAGTACGTGATCGTAGCTCGCTCACACACCCTTTGTCGGATGAGTTTAACGACTGAGTAGTTAGCTGTCAGTTGACTTTTTTTTTTTCTCTCGATATAATTTTAATGGGATGTTGACTTTCTTTTTTTGAATTTTTTTTGTTCATGGCCCATCGACAAATGATCGAATGCATAGTCTGAGTGACCCGGCGTTTTAATGCTGCACCCTTGTTCCCCAAGCAAATTATGTTACACTAACGTCAATGTTGCCTAGTTAAGTGGTATATCTGCTCATTTTATTTCAGAAACGAATTAAGCAACC
