# Supplementary material for: Exploring the Use of Alternative Promoters for Enhanced Transgene and sgRNA Expression in Atlantic Salmon Cells
Source: Mar Biotechnol (NY). 2024 Aug 30;26(6):1143–54. doi: 10.1007/s10126-024-10362-4 (PMC11541246; doi:10.1007/s10126-024-10362-4)
Supplement: Supplementary file 6 — Supplementary file6 (DOCX 16 KB) [file 10126_2024_10362_MOESM6_ESM.docx]

Supplementary sequences 2: Potential salmon U6 promoters in the salmon genome. The sequences is immediately preceding spliceasomal RNA.

>USEDSSU6

AGTGTACTTGCATATCACCCAGCATACATTGCAACTTTCTGAGGCAGAGGCTAGAACATTACAACACACAAGGTAGCCATTAAACGATACATTATTATCCCTACCAGGTTCATGTCTATACAATATCCTGTGGGAATCTTCTATGGGTGGTTTTTGAGCCTCTGAAGGTCTGTTTGACCCCAACCAGGATCCGAAACGTCAGGGGGGTTGTGCTCTATATATGAGGGCCTCTCCTATGTCTGTATT

>LOC123743874

AGGGGGAAACCACACCAGTCACTCTACCGCTACTCTACATAACCCCCAGCATGCCCTACTGCTCGGTTAAAGGCAACTGGTAGAAGCTTTTAGATGGTAACTCCGAGAGCTCTGTGTGTGTATATAGATGTGTTGATTGTTTTAAGCATGCGTGAGTCTTATCAGGGTGTTTCCAGAGTATAACCGTTAATGAGACTGCTAATCTCGACTGGTCCTACAAATAATGTCTGATCAGAGTGACTATTT

>LOC123740243

GCCAGGCCATGTTGCTGAAAATGGGGGAAGGGTGGGAAGTGGTCGGGGCAGAGGAACAAAGACTTGGGGAGGAGCAACTTAAAGCAGCAGGTGAGGGGTTAGAGACCAAGAGCCCTGAATCATAAAAGGCTTAGGGTAGTTTTTGAGAGTCTGAAGGTCAGTTTGAACCCTGTTTGACCCCAACCATGACCAGAACCATCAGAGGGGTTGTGTTCTATATATGAGGGCCTCTCTTAGGTTTGTATT

>LOC123740238

GCCAGGCCATGTTGCAGAAAATGGGGGAAGGGTGGGAAGTGGTCGGGGCAGAGGAACAAAGACTTGGGGAGGAGCAACTTAAAGCAGCAGGTGAGGGGTTAGAGACCAAGAGCCCTGAATCATAAAAGGCTTAGGGTAGTTTTTGAGAGTCTGAAGGTCAGTTTGAACCCTGTTTGACCCCAACCATGACCAGAACCATCAGAGGGGTTGTGTTCTATATATGAGGGCCTCTCTTAGGTTTGTATT

>LOC123740237

GCCAGGCCATGTTGCAGAAAATGGGGGAAGGGTGGGAAGTGGTCGGGGCAGAGGAACAAAGACTTGGGGAGGAGCAACTTAAAGCAGCAGGTGAGGGGTTAGAGACCAAGAGCCCTGAATCATAAAAGGCTTAGGGTAGTTTTTGAGAGTCTGAAGGTCAGTTTGAACCCTGTTTGACCCCAACCATGACCAGAACCATCAGAGGGGTTGTGTTCTATATATGAGGGCCTCTCTTAGGTTTGTATT

>LOC123740235

GCCAGGCCATGTTGCAGAAAATGGGGGAAGGGTGGGAAGTGGTCGGGGCAGAGGAACAAAGACTTGGGGAGGAGCAACTTAAAGCAGCAGGTGAGGGGTTAGAGACCAAGAGCCCTGAATCATAAAAGGCTTAGGGTAGTTTTTGAGAGTCTGAAGGTCAGTTTGAACCCTGTTTGACCCCAACCATGACCAGAACCATCAGAGGGGTTGTGTTCTATATATGAGGGCCTCTCTTAGGTTTGTATT

>LOC123740231

GCCAGGCCATGTTGCAGAAAATGGGGGAAGGGTGGGAAGTGGTCGGGGCAGAGGAACAAAGACTTGGGGAGGAGCAACTTAAAGCAGCAGGTGAGGGGTTAGAGACCAAGAGCCCTGAATCATAAAAGGCTTAGGGTAGTTTTTGAGAGTCTGAAGGTCAGTTTGAACCCTGTTTGACCCCAACCATGACCAGAACCATCAGAGGGGTTGTGTTCTATATATGAGGGCCTCTCTTAGGTTTGTATT

>LOC123740228

GCCAGGCCATGTTGCAGAAAATGGGGGAAGGGTGGGAAGTGGTCGGGGCAGAGGAACAAAGACTTGGGGAGGAGCAACTTAAAGCAGCAGGTGAGGGGTTAGAGACCAAGAGCCCTGAATCATAAAAGGCTTAGGGTAGTTTTTGAGAGTCTGAAGGTCAGTTTGAACCCTGTTTGACCCCAACCATGACCAGAACCATCAGAGGGGTTGTGTTCTATATATGAGGGCCTCTCTTAGGTTTGTATT

>LOC123740226

GCCAGGCCATGTTGCAGAAAATGGGGGAAGGGTGGGAAGTGGTCGGGGCAGAGGAACAAAGACTTGGGGAGGAGCAACTTAAAGCAGCAGGTGAGGGGTTAGAGACCAAGAGCCCTGAATCATAAAAGGCTTAGGGTAGTTTTTGAGAGTCTGAAGGTCAGTTTGAACCCTGTTTGACCCCAACCATGACCAGAACCATCAGAGGGGTTGTGTTCTATATATGAGGGCCTCTCTTAGGTTTGTATT

>LOC123740223

CCAGGCCATGTTGCAGAAAATGGGGGAAGGGTGGGAAGTGGTCGGGGCAGAGGAACAAAGACTTGGGGAGGAGCAACTTAAAGCAGCAGGTGAGGGGTTAGAGACCAAGAGCCCTGAATCATAAAAAGGCTTAGGGTAGTTTTTGAGAGTCTGAAGGTCAGTTTGAACCCTGTTTGACCCCAACCATGACCAGAACCATCAGAGGGGTTGTGTTCTATATATGAGGGCCTCTCTTAGGTTTGTATT

>LOC123740216

GCCAGGCCATGTTGCAGAAAATGGGGGAAGGGTGGGAAGTGGTCGGGGCAGAGGAACAAAGACTTGGGGAGGAGCAACTTAAAGCAGCAGGTGAGGGGTTAGAGACCAAGAGCCCTGAATCATAAAAGGCTTAGGGTAGTTTTTGAGAGTCTGAAGGTCAGTTTGAACCCTGTTTGACCCCAACCATGACCAGAACCATCAGAGGGGTTGTGTTCTATATATGAGGGCCTCTCTTAGGTTTGTATT

>LOC123740205

GCCAGGCCATGTTGCTGAAAATGGGGGAAGGGTGGGAAGTGGTCGGGGCAGAGGAACAAAGACTTGGGGAGGAGCAAGTTAAAGCAGCAGGTGAGGGGTTAGAGACCAAGAGCCCTGAATCATAAAAGGCTTAGGGTAGTTTTTGAGAGTCTGAAGGTCAGTTTGAACCCTGTTTGACCCCAACCATGACCAGAACCATCAGAGGGGTTGTGTTCTATATATGAGGGCCTCTCTTAGGTTTGTATT

>LOC123740194

TGCCAGGCCATGTTGCAGAAAATGGGGAAGGGTGGGAAGTGGTCGGGGCAGAGGAACAAAGACTTGGGGAGGAGCAACTTAAAGCAGCAGGTGAGGGGTTAGAGACCAAGAGCCCTGAATCATAAAAGGCTTAGGGTAGTTTTTGAGAGTCTGAAGGTCAGTTTGAACCCTGTTTGACCCCAACCATGACCAGAACCATCAGAGGGGTTGTGTTCTATATATGAGGGCCTCTCTTAGGTTTGTATT

>LOC123740183

GCCAGGCCATGTTGCAGAAAATGGGGGAAGGGTGGGAAGTGGTCGGGGCAGAGGAACAAAGACTTGGGGAGGAGCAACTTAAAGCAGCAGGTGAGGGGTTAGAGACCAAGAGCCCTGAATCATAAAAGGCTTAGGGTAGTTTTTGAGAGTCTGAAGGTCAGTTTGAACCCTGTTTGACCCCAACCATGACCAGAACCATCAGAGGGGTTGTGTTCTATATATGAGGGCCTCTCTTAGGTTTGTATT

>LOC123740172

GCCAGGCCATGTTGCAGAAAATGGGGGAAGGGTGGGAAGTGGTCGGGGCAGAGGAACAAAGACTTGGGGAGGAGCAACTTAAAGCAGCAGGTGAGGGGTTAGAGACCAAGAGCCCTGAATCATAAAAGGCTTAGGGTAGTTTTTGAGAGTCTGAAGGTCAGTTTGAACCCTGTTTGACCCCAACCATGACCAGAACCATCAGAGGGGTTGTGTTCTATATATGAGGGCCTCTCTTAGGTTTGTATT

>LOC123740161

TGCCAGGCCATGTTGCAGAAAATGGGGAAGGGTGGGAAGTGGTCGGGGCAGAGGAACAAAGACTTGGGGAGGAGCAACTTAAAGCAGCAGGTGAGGGGTTAGAGACCAAGAGCCCTGAATCATAAAAGGCTTAGGGTAGTTTTTGAGAGTCTGAAGGTCAGTTTGAACCCTGTTTGACCCCAACCATGACCAGAACCATCAGAGGGGTTGTGTTCTATATATGAGGGCCTCTCTTAGGTTTGTATT

>LOC123740150

GCCAGGCCATGTTGCAGAAAATGGGGGAAGGGTGGGAAGTGGTCGGGGCAGAGGAACAAAGACTTGGGGAGGAGCAACTTAAAGCAGCAGGTGAGGGGTTAGAGACCAAGAGCCCTGAATCATAAAAGGCTTAGGGTAGTTTTTGAGAGTCTGAAGGTCAGTTTGAACCCTGTTTGACCCCAACCATGACCAGAACCATCAGAGGGGTTGTGTTCTATATATGAGGGCCTCTCTTAGGTTTGTATT

>LOC123740139

TGCCAGGCCATGTTGCAGAAAATGGGGAAGGGTGGGAAGTGGTCGGGGCAGAGGAACAAAGACTTGGGGAGGAGCAACTTAAAGCAGCAGGTGAGGGGTTAGAGACCAAGAGCCCTGAATCATAAAAGGCTTAGGGTAGTTTTTGAGAGTCTGAAGGTCAGTTTGAACCCTGTTTGACCCCAACCATGACCAGAACCATCAGAGGGGTTGTGTTCTATATATGAGGGCCTCTCTTAGGTTTGTATT

>LOC123740128

TGCCAGGCCATGTTGCAGAAAATGGGGAAGGGTGGGAAGTGGTCGGGGCAGAGGAACAAAGACTTGGGGAGGAGCAACTTAAAGCAGCAGGTGAGGGGTTAGAGACCAAGAGCCCTGAATCATAAAAGGCTTAGGGTAGTTTTTGAGAGTCTGAAGGTCAGTTTGAACCCTGTTTGACCCCAACCATGACCAGAACCATCAGAGGGGTTGTGTTCTATATATGAGGGCCTCTCTTAGGTTTGTATT

>LOC123740118

GCCAGGCCATGTTGCAGAAAATGGGGGAAGGGTGGGAAGTGGTCGGGGCAGAGGAACAAAGACTTGGGGAGGAGCAACTTAAAGCAGCAGGTGAGGGGTTAGAGACCAAGAGCCCTGAATCATAAAAGGCTTAGGGTAGTTTTTGAGAGTCTGAAGGTCAGTTTGAACCCTGTTTGACCCCAACCATGACCAGAACCATCAGAGGGGTTGTGTTCTATATATGAGGGCCTCTCTTAGGTTTGTATT

>LOC123740108

GCCAGGCCATGTTGCAGAAAATGGGGGAAGGGTGGGAAGTGGTCGGGGCAGAGGAACAAAGACTTGGGGAGGAGCAACTTAAAGCAGCAGGTGAGGGGTTAGAGACCAAGAGCCCTGAATCATAAAAGGCTTAGGGTAGTTTTTGAGAGTCTGAAGGTCAGTTTGAACCCTGTTTGACCCCAACCATGACCAGAACCATCAGAGGGGTTGTGTTCTATATATGAGGGCCTCTCTTAGGTTTGTATT

>LOC123740097

GCCAGGCCATGTTGCAGAAAATGGGGGAAGGGTGGGAAGTGGTCGGGGCAGAGGAACAAAGACTTGGGGAGGAGCAACTTAAAGCAGCAGGTGAGGGGTTAGAGACCAAGAGCCCTGAATCATAAAAGGCTTAGGGTAGTTTTTGAGAGTCTGAAGGTCAGTTTGAACCCTGTTTGACCCCAACCATGACCAGAACCATCAGAGGGGTTGTGTTCTATATATGAGGGCCTCTCTTAGGTTTGTATT

>LOC123740078

GCCAGGCCATGTTGCTGAAAATGGGGGAAGGGTGGGAAGTGGTCGGGGCAGAGGAACAAAGACTTGGGGAGGAGCAACTTAAAGCAGCAGGTGAGGGGTTAGAGACCAAGAGCCCTGAATCATAAAAGGCTTAGGGTAGTTTTTGAGAGTCTGAAGGTCAGTTTGAACCCTGTTTGACCCCAACCATGACCAGAACCATCAGAGGGGTTGTGTTCTATATATGAGGGCCTCTCTTAGGTTTGTATT

>LOC123740071

GCCAGGCCATGTTGCAGAAAATGGGGGAAGGGTGGGAAGTGGTCGGGGCAGAGGAACAAAGACTTGGGGAGGAGCAACTTAAAGCAGCAGGTGAGGGGTTAGAGACCAAGAGCCCTGAATCATAAAAGGCTTAGGGTAGTTTTTGAGAGTCTGAAGGTCAGTTTGAACCCTGTTTGACCCCAACCATGACCAGAACCATCAGAGGGGTTGTGTTCTATATATGAGGGCCTCTCTTAGGTTTGTATT

>LOC123740065

GCCAGGCCATGTTGCTGAAAATGGGGGAAGGGTGGGAAGTGGTCGGGGCAGAGGAACAAAGACTTGGGGAGGAGCAACTTAAAGCAGCAGGTGAGGGGTTAGAGACCAAGAGCCCTGAATCATAAAAGGCTTAGGGTAGTTTTTGAGAGTCTGAAGGTCAGTTTGAACCCTGTTTGACCCCAACCATGACCAGAACCATCAGAGGGGTTGTGTTCTATATATGAGGGCCTCTCTTAGGTTTGTATT

>LOC123740064

GCCAGGCCATGTTGCTGAAAATGGGGGAAGGGTGGGAAGTGGTCGGGGCAGAGGAACAAAGACTTGGGGAGGAGCAACTTAAAGCAGCAGGTGAGGGGTTAGAGACCAAGAGCCCTGAATCATAAAAGGCTTAGGGTAGTTTTTGAGAGTCTGAAGGTCAGTTTGAACCCTGTTTGACCCCAACCATGACCAGAACCATCAGAGGGGTTGTGTTCTATATATGAGGGCCTCTCTTAGGTTTGTATT

>LOC123740062

GCCAGGCCATGTTGCTGAAAATGGGGGAAGGGTGGGAAGTGGTCGGGGCAGAGGAACAAAGACTTGGGGAGGAGCAACTTAAAGCAGCAGGTGAGGGGTTAGAGACCAAGAGCCCTGAATCATAAAAGGCTTAGGGTAGTTTTTGAGAGTCTGAAGGTCAGTTTGAACCCTGTTTGACCCCAACCATGACCAGAACCATCAGAGGGGTTGTGTTCTATATATGAGGGCCTCTCTTAGGTTTGTATT

>LOC123740051

GCCAGGCCATGTTGCAGAAAATGGGGGAAGGGTGGGAAGTGGTCGGGGCAGAGGAACAAAGACTTGGGGAGGAGCAACTTAAAGCAGCAGGTGAGGGGTTAGAGACCAAGAGCCCTGAATCATAAAAGGCTTAGGGTAGTTTTTGAGAGTCTGAAGGTCAGTTTGAACCCTGTTTGACCCCAACCATGACCAGAACCATCAGAGGGGTTGTGTTCTATATATGAGGGCCTCTCTTAGGTTTGTATT

>LOC123740040

TGCCAGGCCATGTTGCTGAAAATGGGGAAGGGTGGGAAGTGGTCGGGGCAGAGGAACAAAGACTTGGGGAGGAGCAACTTAAAGCAGCAGGTGAGGGGTTAGAGACCAAGAGCCCTGAATCATAAAAGGCTTAGGGTAGTTTTTGAGAGTCTGAAGGTCAGTTTGAACCCTGTTTGACCCCAACCATGACCAGAACCATCAGAGGGGTTGTGTTCTATATATGAGGGCCTCTCTTAGGTTTGTATT

>LOC123740031

GCCAGGCCATGTTGCTGAAAATGGGGGAAGGGTGGGAAGTGGTCGGGGCAGAGGAACAAAGACTTGGGGAGGAGCAACTTAAAGCAGCAGGTGAGGGGTTAGAGACCAAGAGCCCTGAATCATAAAAGGCTTAGGGTAGTTTTTGAGAGTCTGAAGGTCAGTTTGAACCCTGTTTGACCCCAACCATGACCAGAACCATCAGAGGGGTTGTGTTCTATATATGAGGGCCTCTCTTAGGTTTGTATT

>LOC123740025

GCCAGGCCATGTTGCTGAAAATGGGGGAAGGGTGGGAAGTGGTCGGGGCAGAGGAACAAAGACTTGGGGAGGAGCAAGTTAAAGCAGCAGGTGAGGGGTTAGAGACCAAGAGCCCTGAATCATAAAAGGCTTAGGGTAGTTTTTGAGAGTCTGAAGGTCAGTTTGAACCCTGTTTGACCCCAACCATGACCAGAACCATCAGAGGGGTTGTGTTCTATATATGAGGGCCTCTCTTAGGTTTGTATT

>LOC123740022

GCCAGGCCATGTTGCTGAAAATGGGGGAAGGGTGGGAAGTGGTCGGGGCAGAGGAACAAAGACTTGGGGAGGAGCAACTTAAAGCAGCAGGTGAGGGGTTAGAGACCAAGAGCCCTGAATCATAAAAGGCTTAGGGTAGTTTTTGAGAGTCTGAAGGTCAGTTTGAACCCTGTTTGACCCCAACCATGACCAGAACCATCAGAGGGGTTGTGTTCTATATATGAGGGCCTCTCTTAGGTTTGTATT

>LOC123740009

GCCAGGCCATGTTGCTGAAAATGGGGGAAGGGTGGGAAGTGGTCGGGGCAGAGGAACAAAGACTTGGGGAGGAGCAACTTAAAGCAGCAGGTGAGGGGTTAGAGACCAAGAGCCCTGAATCATAAAAGGCTTAGGGTAGTTTTTGAGAGTCTGAAGGTCAGTTTGAACCCTGTTTGACCCCAACCATGACCAGAACCATCAGAGGGGTTGTGTTCTATATATGAGGGCCTCTCTTAGGTTTGTATT

>LOC123740002

GCCAGGCCATGTTGCTGAAAATGGGGGAAGGGTGGGAAGTGGTCGGGGCAGAGGAACAAAGACTTGGGGAGGAGCAAGTTAAAGCAGCAGGTGAGGGGTTAGAGACCAAGAGCCCTGAATCATAAAAGGCTTAGGGTAGTTTTTGAGAGTCTGAAGGTCAGTTTGAACCCTGTTTGACCCCAACCATGACCAGAACCATCAGAGGGGTTGTGTTCTATATATGAGGGCCTCTCTTAGGTTTGTATT

>LOC123739992

GCCAGGCCATGTTGCTGAAAATGGGGGAAGGGTGGGAAGTGGTCGGGGCAGAGGAACAAAGACTTGGGGAGGAGCAACTTAAAGCAGCAGGTGAGGGGTTAGAGACCAAGAGCCCTGAATCATAAAAGGCTTAGGGTAGTTTTTGAGAGTCTGAAGGTCAGTTTGAACCCTGTTTGACCCCAACCATGACCAGAACCATCAGAGGGGTTGTGTTCTATATATGAGGGCCTCTCTTAGGTTTGTATT

>LOC123739981

GCCAGGCCATGTTGCTGAAAATGGGGGAAGGGTGGGAAGTGGTCGGGGCAGAGGAACAAAGACTTGGGGAGGAGCAACTTAAAGCAGCAGGTGAGGGGTTAGAGACCAAGAGCCCTGAATCATAAAAGGCTTAGGGTAGTTTTTGAGAGTCTGAAGGTCAGTTTGAACCCTGTTTGACCCCAACCATGACCAGAACCATCAGAGGGGTTGTGTTCTATATATGAGGGCCTCTCTTAGGTTTGTATT

>LOC123739970

GCCAGGCCATGTTGCTGAAAATGGGGGAAGGGTGGGAAGTGGTCGGGGCAGAGGAACAAAGACTTGGGGAGGAGCAACTTAAAGCAGCAGGTGAGGGGTTAGAGACCAAGAGCCCTGAATCATAAAAGGCTTAGGGTAGTTTTTGAGAGTCTGAAGGTCAGTTTGAACCCTGTTTGACCCCAACCATGACCAGAACCATCAGAGGGGTTGTGTTCTATATATGAGGGCCTCTCTTAGGTTTGTATT

>LOC123739959

GCCAGGCCATGTTGCTGAAAATGGGGGAAGGGTGGGAAGTGGTCGGGGCAGAGGAACAAAGACTTGGGGAGGAGCAAGTTAAAGCAGCAGGTGAGGGGTTAGAGACCAAGAGCCCTGAATCATAAAAGGCTTAGGGTAGTTTTTGAGAGTCTGAAGGTCAGTTTGAACCCTGTTTGACCCCAACCATGACCAGAACCATCAGAGGGGTTGTGTTCTATATATGAGGGCCTCTCTTAGGTTTGTATT

>LOC123739948

GCCAGGCCATGTTGCTGAAAATGGGGGAAGGGTGGGAAGTGGTCGGGGCAGAGGAACAAAGACTTGGGGAGGAGCAACTTAAAGCAGCAGGTGAGGGGTTAGAGACCAAGAGCCCTGAATCATAAAAGGCTTAGGGTAGTTTTTGAGAGTCTGAAGGTCAGTTTGAACCCTGTTTGACCCCAACCATGACCAGAACCATCAGAGGGGTTGTGTTCTATATATGAGGGCCTCTCTTAGGTTTGTATT

>LOC123739926

GCCAGGCCATGTTGCTGAAAATGGGGGAAGGGTGGGAAGTGGTCGGGGCAGAGGAACAAAGACTTGGGGAGGAGCAACTTAAAGCAGCAGGTGAGGGGTTAGAGACCAAGAGCCCTGAATCATAAAAGGCTTAGGGTAGTTTTTGAGAGTCTGAAGGTCAGTTTGAACCCTGTTTGACCCCAACCATGACCAGAACCATCAGAGGGGTTGTGTTCTATATATGAGGGCCTCTCTTAGGTTTGTATT

>LOC123739838

GCCAGGCCATGTTGCTGAAAATGGGGGAAGGGTGGGAAGTGGTCGGGGCAGAGGAACAAAGACTTGGGGAGGAGCAACTTAAAGCAGCAGGTGAGGGGTTAGAGACCAAGAGCCCTGAATCATAAAAGGCTTAGGGTAGTTTTTGAGAGTCTGAAGGTCAGTTTGAACCCTGTTTGACCCCAACCATGACCAGAACCATCAGAGGGGTTGTGTTCTATATATGAGGGCCTCTCTTAGGTTTGTATT

>LOC123739717

GCCAGGCCATGTTGCTGAAAATGGGGGAAGGGTGGGAAGTGGTCGGGGCAGAGGAACAAAGACTTGGGGAGGAGCAACTTAAAGCAGCAGGTGAGGGGTTAGAGACCAAGAGCCCTGAATCATAAAAGGCTTAGGGTAGTTTTTGAGAGTCTGAAGGTCAGTTTGAACCCTGTTTGACCCCAACCATGACCAGAACCATCAGAGGGGTTGTGTTCTATATATGAGGGCCTCTCTTAGGTTTGTATT

>LOC123732775

TGCCAGGCCATGTTGCTGAAAATGGGGAAGGGTGGGAAGTGGTCGGGGCAGAGGAACAAAGACTTGGGGAGGAGCAACTTAAAGCAGCAGGTGAGGGGTTAGAGACCAAGAGCCCTGAATCATAAAAGGCTTAGGGTAGTTTTTGAGAGTCTGAAGGTCAGTTTGAACCCTGTTTGACCCCAACCATGACCAGAACCATCAGAGGGGTTGTGTTCTATATATGAGGGCCTCTCTTAGGTTTGTATT

>LOC123732774

GCCAGGCCATGTTGCTGAAAATGGGGGAAGGGTGGGAAGTGGTCGGGGCAGAGGAACAAAGACTTGGGGAGGAGCAACTTAAAGCAGCAGGTGAGGGGTTAGAGACCAAGAGCCCTGAATCATAAAAGGCTTAGGGTAGTTTTTGAGAGTCTGAAGGTCAGTTTGAACCCTGTTTGACCCCAACCATGACCAGAACCATCAGAGGGGTTGTGTTCTATATATGAGGGCCTCTCTTAGGTTTGTATT

>LOC123732773

TGCCAGGCCATGTTGCTGAAAATGGGGAAGGGTGGGAAGTGGTCGGGGCAGAGGAACAAAGACTTGGGGAGGAGCAACTTAAAGCAGCAGGTGAGGGGTTAGAGACCAAGAGCCCTGAATCATAAAAGGCTTAGGGTAGTTTTTGAGAGTCTGAAGGTCAGTTTGAACCCTGTTTGACCCCAACCATGACCAGAACCATCAGAGGGGTTGTGTTCTATATATGAGGGCCTCTCTTAGGTTTGTATT

>LOC123732772

GCCAGGCCATGTTGCTGAAAATGGGGGAAGGGTGGGAAGTGGTCGGGGCAGAGGAACAAAGACTTGGGGAGGAGCAACTTAAAGCAGCAGGTGAGGGGTTAGAGACCAAGAGCCCTGAATCATAAAAGGCTTAGGGTAGTTTTTGAGAGTCTGAAGGTCAGTTTGAACCCTGTTTGACCCCAACCATGACCAGAACCATCAGAGGGGTTGTGTTCTATATATGAGGGCCTCTCTTAGGTTTGTATT

>LOC123732771

GCCAGGCCATGTTGCTGAAAATGGGGGAAGGGTGGGAAGTGGTCGGGGCAGAGGAACAAAGACTTGGGGAGGAGCAACTTAAAGCAGCAGGTGAGGGGTTAGAGACCAAGAGCCCTGAATCATAAAAGGCTTAGGGTAGTTTTTGAGAGTCTGAAGGTCAGTTTGAACCCTGTTTGACCCCAACCATGACCAGAACCATCAGAGGGGTTGTGTTCTATATATGAGGGCCTCTCTTAGGTTTGTATT

>LOC123732770

GCCAGGCCATGTTGCTGAAAATGGGGGAAGGGTGGGAAGTGGTCGGGGCAGAGGAACAAAGACTTGGGGAGGAGCAACTTAAAGCAGCAGGTGAGGGGTTAGAGACCAAGAGCCCTGAATCATAAAAGGCTTAGGGTAGTTTTTGAGAGTCTGAAGGTCAGTTTGAACCCTGTTTGACCCCAACCATGACCAGAACCATCAGAGGGGTTGTGTTCTATATATGAGGGCCTCTCTTAGGTTTGTATT

>LOC123732769

GCCAGGCCATGTTGCTGAAAATGGGGGAAGGGTGGGAAGTGGTCGGGGCAGAGGAACAAAGACTTGGGGAGGAGCAACTTAAAGCAGCAGGTGAGGGGTTAGAGACCAAGAGCCCTGAATCATAAAAGGCTTAGGGTAGTTTTTGAGAGTCTGAAGGTCAGTTTGAACCCTGTTTGACCCCAACCATGACCAGAACCATCAGAGGGGTTGTGTTCTATATATGAGGGCCTCTCTTAGGTTTGTTTT

>LOC123731970

TGCCAGGCCATGTTGCAGAAAATGGGGAAGGGTGGGAAGTGGTCGGGGCAGAGGAACAAAGACTTGGGGAGGAGCAACTTAAAGCAGCAGGTGAGGGGTTAGAGACCAAGAGCCCTGAATCATAAAAGGCTTAGGGTAGTTTTTGAGAGTCTGAAGGTCAGTTTGAACCCTGTTTGACCCCAACTATGACCAGAACCATCAGAGGGGTTGTGTTCTATTTATGAGGGCCTCTCTTAGGTTTGTATT

>LOC123731969

GCCAGGCCATGTTGCAGAAAATGGGGGAAGGGTGGGAAGTGGTCGGGGCAGAGGAACAAAGACTTGGGGAGGAGCAACTTAAAGCAGCAGGTGAGGGGTTAGAGACCAAGAGCCCTGAATCATAAAAGGCTTAGGGTAGTTTTTGAGAGTCTGAAGGTCAGTTTGAACCCTGTTTGACCCCAACTATGACCAGAACCATCAGAGGGGTTGTGTTCTATTTATGAGGGCCTCTCTTAGGTTTGTATT

>LOC123731968

CCAGGCCATGTTGCAGAAAATGGGGGAAGGGTGGGAAGTGGTCGGGGCAGAGGAACAAAGACTTGGGGAGGAGCAACTTAAAGCAGCAGGTGAGGGGTTAGAGACCAAGAGCCCTGAATCATAAAAGGCTTAGGGTAGTTTTTGAGAGTCTGAAGGTCAGTTTGAACCCTGTTTGACCCCAACTATGACCAGAACCATCAGAGGGGTTGTGTTCTATTTATGAGGGCCTCTCTTAGGGTTTGTATT

>LOC123731967

TGCCAGGCCATGTTGCTGAAAATGGGGAAGGGTGGGAAGTGGTCGGGGCAGAGGAACAAAGACTTGGGGAGGAGCAACTTAAAGCAGCAGGTGAGGGGTTAGAGACCAAGAGCCCTGAATCATAAAAGGCTTAGGGTAGTTTTTGAGAGTCTGAAGGTCAGTTTGAACCCTGTTTGACCCCAACCATGACCAGAACCATCAGAGGGGTTGTGTTCTATATATGAGGGCCTCTCTTAGGTTTGTATT

>LOC123731963

GCCAGGCCATGTTGCAGAAAATGGGGGAAGGGTGGGAAGTGGTCGGGGCAGAGGAACAAAGACTTGGGGAGGAGCAACTTAAAGCAGCAGGTGAGGGGTTAGAGACCAAGAGCCCTGAATCATAAAAGGCTTAGGGTAGTTTTTGAGAGTCTGAAGGTCAGTTTGAACCCTGTTTGACCCCAACCATGACCAGAACCATCAGAGGGGTTGTGTTCTATATATGAGGGCCTCTCTTAGGTTTGTATT

>LOC123731962

GCCAGGCCATGTTGCAGAAAATGGGGGAAGGGTGGGAAGTGGTCGGGGCAGAGGAACAAAGACTTGGGGAGGAGCAACTTAAAGCAGCAGGTGAGGGGTTAGAGACCAAGAGCCCTGAATCATAAAAGGCTTAGGGTAGTTTTTGAGAGTCTGAAGGTCAGTTTGAACCCTGTTTGACCCCAACCATGACCAGAACCATCAGAGGGGTTGTGTTCTATATATGAGGGCCTCTCTTAGGTTTGTATT

>LOC123731961

GCCAGGCCATGTTGCAGAAAATGGGGAAAGGGTGGGAAGTGGTCGGGGCAGAGGAACAAAGACTTGGGGAGGAGCAACTTAAAGCAGCAGGTGAGGGGTTAGAGACCAAGAGCCCTGAATCATAAAAGGCTTAGGGTAGTTTTTGAGAGTCTGAAGGTCAGTTTGAACCCTGTTTGACCCCAACCATGACCAGAACCATCAGAGGGGTTGTGTTCTATATATGAGGGCCTCTCTTAGGTTTGTATT

>LOC123731960

GCCAGGCCATGTTGCAGAAAATGGGGAAGGGTGGGAAGTGGTCGGGGCAGAGGAACAAAGACTTGGGGAGGAGCAACTTAAAGCAGCAGGTGAGGGGTTAGAGACCAAGAGCCCTGAATCATAAAAAGGCTTAGGGTAGTTTTTGAGAGTCTGAAGGTCAGTTTGAACCCTGTTTGACCCCAACTATGACCAGAACCATCAGAGGGGTTGTGTTCTATTTATGAGGGCCTCTCTTAGGTTTGTATT

>LOC123731959

CCAGGCCATGTTGCAGAAAATGGGGGAAGGGTGGGAAGTGGTCGGGGCAGAGGAACAAAGACTTGGGGAGGAGCAACTTAAAGCAGCAGGTGAGGGGTTAGAGACCAAGAGCCCTGAATCATAAAAAGGCTTAGGGTAGTTTTTGAGAGTCTGAAGGTCAGTTTGAACCCTGTTTGACCCCAACCATGACCAGAACCATCAGAGGGGTTGTGTTCTATATATGAGGGCCTCTCTTAGGTTTGTATT

>LOC123731956

GCCAGACCATGTTGCAGAAAATGGGGGAAGGGTGGGAAGTGGTCGGGGCAGAGGAACAAAGACTTGGGGAGGAGCAACTTAAAGCAGCAGGTGAGGGGTTAGAGACCAAGAGCCCTGAATCATAAAAGGCTTAGGGTAGTTTTTGAGAGTCTGAAGGTCAGTTTGAACCCTGTTTGACCCCAACCATGACCAGAACCATCAGAGGGGTTGTGTTCTATATATGAGGGCCTCTCTTAGGTTTGTATT

>LOC123731955

AATCAAACAATTCAGTTGGCTAGACGTGTTATAGTCATATTTCATCACATTTATAGTCATTGATGTTAACCTGTTGGGGGTAGGGGGCAGTATTTGCACGGCCGGATAAAAAACGTAATCTGGTTATTACTACTGCCCAGAAACTAGAATATGAAGGTCAGTTTGAACCCTGTTTGACCCCAACCATGACCAGAACCATCAGAGGGGTTGTGTTCTATATATGAGGGCCTCTCTTAGGTTTGTATT

>LOC123731954

GCCAGGCCATGTTGCAGAAAATGGGGGAAGGGTGGGAAGTGGTCGGGGCAGAGGAACAAAGACTTGGGGAGGAGCAACTTAAAGCAGCAGGTGAGGGGTTAGAGACCAAGAGCCCTGAATCATAAAAGGCTTAGGGTAGTTTTTGAGAGTCTGAAGGTCAGTTTGAACCCTGTTTGACCCCAACCATGACCAGAACCATCAGAGGGGTTGTGTTCTATATATGAGGGCCTCTCTTAGGTTTGTATT

>LOC123725760

TCCTAGACGGGACAGTGTACTCGCATATCACCCAGCATACATTGCAACTCTCTGAGGCAGAGGCTAGAACATTACAACACACAAGGTAGCCATTATTATCCCAACCAGGTTCATGTCCATACAATATCCTGTGGGAATCTTCTATGGGTGGTTTTTGAGCCTCTGAAGGTCTGTTTGACCCCAACCAGGATCCGAAACGTCAGGGGGTTGTGCTCTATATATGAGGGCCTCTCCTATGTCTGTATT

>LOC123725759

CCTAGACGGGACAGTGTACTCGCATATCACCCAGCATACATTGCAACTCTCTGAGGCAGAGGCTAGAACATTACAACACACAAGGTAGCCATTATTATCCCAACCAGGTTCATGTCCATACAATATCCTGTGGGAATCTTCTATGGGTGGTTTTTGAGCCTCTGAAGGTCTGTTTGACCCCAACCAGGATCCGAAACGTCAGGGGGGTTGTGCTCTATATATGAGGGCCTCTCCTATGTCTGTATT

>LOC123725757

CCTAGACGGGACAGTGTACTCGCATATCACCCAGCATACATTGCAACTCTCTGAGGCAGAGGCTAGAACATTACAACACACAAGGTAGCCATTATTATCCCAACCAGGTTCATGTCCATACAATATCCTGTGGGAATCTTCTATGGGTGGTTTTTGAGCCTCTGAAGGTCTGTTTGACCCCAACCAGGATCCGAAACGTCAGGGGGGTTGTGCTCTATATATGAGGGCCTCTCCTATGTCTGTATT

>LOC123725756

GTCCTAGACGGGACAGTGTACTCGCATATCACCCAGCATACATTGCAACTCTCTGAGGCAGAGGCTAGAACATTACAACACACAAGGTAGCCATTATTATCCCAACCAGGTTCATGTCCACACAATATCCTGTGGGAATCTTCTATGGGTGGTTTTTTGAGCCTCTGAAGGTCTGTTTGACCCCAACCAGGATCCGAAACGTCAGGGGGTTGTGCCTATATATGAGGGCCTCTCCTATGTCTGTATT
